# An essential vesicular-trafficking phospholipase mediates neutral lipid synthesis and contributes to hemozoin formation in *Plasmodium falciparum*

**DOI:** 10.1186/s12915-021-01042-z

**Published:** 2021-08-11

**Authors:** Mohd Asad, Yoshiki Yamaryo-Botté, Mohammad E. Hossain, Vandana Thakur, Shaifali Jain, Gaurav Datta, Cyrille Y. Botté, Asif Mohmmed

**Affiliations:** 1 International Centre for Genetic Engineering and Biotechnology, New Delhi, 110 067 India; 2 ApicoLipid Team, Institute for Advanced Biosciences, CNRS UMR5309, Université Grenoble Alpes, INSERM U1209, Grenoble, France

**Keywords:** Phospholipid metabolism, Phospholipase, Neutral lipids, Malaria, Host-haemoglobin, Hemozoin

## Abstract

**Background:**

*Plasmodium falciparum* is the pathogen responsible for the most devastating form of human malaria. As it replicates asexually in the erythrocytes of its human host, the parasite feeds on haemoglobin uptaken from these cells. Heme, a toxic by-product of haemoglobin utilization by the parasite, is neutralized into inert hemozoin in the food vacuole of the parasite. Lipid homeostasis and phospholipid metabolism are crucial for this process, as well as for the parasite’s survival and propagation within the host. *P. falciparum* harbours a uniquely large family of phospholipases, which are suggested to play key roles in lipid metabolism and utilization.

**Results:**

Here, we show that one of the parasite phospholipase (*P. falciparum* lysophospholipase, *Pf*LPL1) plays an essential role in lipid homeostasis linked with the haemoglobin degradation and heme conversion pathway. Fluorescence tagging showed that the *Pf*LPL1 in infected blood cells localizes to dynamic vesicular structures that traffic from the host-parasite interface at the parasite periphery, through the cytosol, to get incorporated into a large vesicular lipid rich body next to the food-vacuole. *Pf*LPL1 is shown to harbour enzymatic activity to catabolize phospholipids, and its transient downregulation in the parasite caused a significant reduction of neutral lipids in the food vacuole-associated lipid bodies. This hindered the conversion of heme, originating from host haemoglobin, into the hemozoin, and disrupted the parasite development cycle and parasite growth. Detailed lipidomic analyses of inducible knock-down parasites deciphered the functional role of *Pf*LPL1 in generation of neutral lipid through recycling of phospholipids. Further, exogenous fatty-acids were able to complement downregulation of *Pf*LPL1 to rescue the parasite growth as well as restore hemozoin levels.

**Conclusions:**

We found that the transient downregulation of *Pf*LPL1 in the parasite disrupted lipid homeostasis and caused a reduction in neutral lipids essentially required for heme to hemozoin conversion. Our study suggests a crucial link between phospholipid catabolism and generation of neutral lipids (TAGs) with the host haemoglobin degradation pathway.

**Supplementary Information:**

The online version contains supplementary material available at 10.1186/s12915-021-01042-z.

## Background

Malaria remains a major parasitic disease in the tropical and sub-tropical countries that resulted in ~229 million cases and around 409,000 deaths globally in the year 2019 [1]. Currently, there is no efficient vaccine and the rapid spread of drug resistant parasite strains, even to the front line treatment using artemisinin combinations [2–4], together plead for the identification of new drug-targets and development of new drugs against the disease. Understanding of key metabolic pathways in the parasite that sustain parasite survival within its human host is critical to identify unique and specific targets in the parasites. The parasite development and division during asexual life cycle in host erythrocytes include dramatic modification of host cell membranous structures (i.e. tubulovesicular network, Maurer’s cleft and knobs) and a massive increase of lipid synthesis and membrane biogenesis needed for parasite division and propagation [5, 6]. Indeed, the parasites require a large amount of lipids to generate new membrane-bound compartments for the assembly of future daughter cells [7–9], for cell signalling event, as well as to generate storage lipids, triacylglycerol (TAG), and cholesteryl-esters [5]. In addition, during the asexual cycle, the parasite utilizes host haemoglobin for its growth and development. Detoxification of host haemoglobin derived heme is an essential pathway for parasite survival during the erythrocytic cycle. The neutral lipids (i.e. TAG, DAG, cholesterol, and cholesteryl esters) have been suggested to be closely associated with heme detoxification during asexual intra-erythrocytic development by allowing/catalyzing formation of hemozoin [10, 11].

Establishment of *P. falciparum* intra-erythrocytic infection is associated with a large increase in the neutral lipid and lipid-associated fatty acid (FA) content in infected red blood cells (iRBCs), suggesting an active lipid metabolism pathway in *P. falciparum* [12]. Furthermore, the phospholipids in an infected red blood cell increase up to 500–700% compared to an uninfected erythrocyte. The phospholipid synthesis machinery is present and highly active in the parasite [5] and is initiated by the synthesis of fatty acids (FA), the building blocks and hydrophobic moieties of most membrane and storage lipids. *P. falciparum* possesses a relict non-photosynthetic plastid, the apicoplast, that harbours the pathway for de novo biosynthesis of fatty acid (FA). This pathway is not essential for asexual stages of the parasite in regular culture conditions [7, 9], but it can be re-activated in adverse lipid composition [6]. Furthermore, the parasite can scavenge a wide range of FA from the host and specifically relies on the import of C16:0 and C18:1 to divide during blood stages [6, 13]. Importantly, the parasite possesses an active de novo phospholipid synthesis machinery (Kennedy pathway, cytidine diphosphate diacylglycerol [CDP-DAG] pathway), capable of assembling and synthesizing all lipid precursors (phosphatidic acid [PA], diacylglycerol [DAG], CDP-DAG) and major phospholipid classes (phosphatidylcholine [PC], phosphatidylethanolamine [PE], phosphatidyl serine [PS], phosphatidylinositol [PI], phosphatidylglycerol [PG], cardiolipin [CL]) from precursors (polar heads, lyso-lipids, FAs) scavenged from the host and its environment [14]. *P. falciparum* blood stages notably rely on scavenging of specific lipid classes/precursors, such as lysophosphatidylcholine (LPC). The utilization of its polar head phosphocholine is reported to fuel the de novo synthesis of the major membrane lipid of the parasite, phosphatidylcholine (PC), via the Kennedy pathway and thus maintain asexual division [14, 15]. Since the Kennedy pathway utilizes the phosphocholine head group from scavenged LPC to synthesize PC, this phosphocholine head group needs to be released from LPC via an undetermined set of enzymes. Taken together, these data indicate that during blood stages, *P. falciparum* relies on the scavenging of phospholipids/FA/lysolipids from the host; however, the molecular machinery that allow to separate and reassemble the phospholipid building blocks (FA, polar heads, lysolipids) remain to be elucidated. Such catabolism of lipid metabolites from the host must involve phospholipases and lysophospholipases to manipulate the scavenged lipids and generate the proper lipid moieties. Phospholipase-like activities are reported from asexual stage parasites, and a phospholipase was shown to play an essential role in the survival of erythrocytic or liver stages of the parasite [16, 17]. *P. falciparum* possesses a large family of lysophospholipases (LPLs), and it is remarkable that most are encoded in the sub-telomeric regions of chromosomes (Plasma DB;[18]); this family also encodes two epoxy-hydrolase like proteins [19], see also Table S1]. Importantly, the biochemical activity of LPL was reported during *P. falciparum* blood stages [20]. The LPLs catalyze the hydrolysis of acyl chains from lysophospholipids, the intermediates in the metabolism of membrane phospholipids, and therefore LPLs play a key role recycling of lipids.

In the present study, we showed that one of the *P. falciparum* lysophospholipase, *Pf*LPL1 plays an essential role in lipid homeostasis linked with the hemozoin formation pathway. We used the degradation domain-based regulated fluorescence tag system [21] for both endogenous localization and transient downregulation of *Pf*LPL1 during the parasite blood stages. The GFP-targeting approach demonstrated that the enzyme is associated with vesicles, which traffic from the parasite periphery (i.e. plasma membrane) towards a compartment accumulating neutral lipids in the vicinity of the food vacuole; the content of neutral lipids in this compartment were dependent upon *Pf*LPL1 levels in the parasite. To further assess the mechanistic details of *Pf*LPL1 role in generation of neutral lipids, we performed detailed lipidomic analyses, which showed that parasites lacking *Pf*LPL1 displayed fluctuations in phospholipid levels, significant reduction of triacylglycerol (TAG), and increased levels of DAGs. Parasite lacking *Pf*LPL1 were found to be specifically deficient in normal generation of hemozoin. Complementation with extracellular fatty acids sources, products of *Pf*LPL1 activity, rescued the hemozoin formation phenotype. Taken together, our data suggest that *Pf*LPL1 is involved in phospholipid catabolism to generate precursors required for neutral lipids (TAGs) synthesis for heme-detoxification, which is an essential pathway for parasite survival.

## Results

### Homologues of *P. falciparum* lysophospholipases

*P. falciparum* genome harbours 13 putative lysophospholipases, which show a high degree of homology among themselves (see supplementary data, Table S1, Figure S1). All the LPLs harbour a GXSXG motif embedding the catalytic active serine (S) that is characteristic of lipases as well as the aspartate (D) and histidine (H) residue forming the catalytic triad [22, 23]. They are ~400 amino acid long, harboring the putative lysophospholipase domain (Hydrolase_4 domain; Pfam 12146) within an alpha-beta hydrolase fold (αβ-hydrolase_1, Pfam00561) of about 200-250 amino acid in length. Two members of this P. falciparum lyso-phospholipase family, PF3D7 1476700 and PF3D7_1476800, also harbor a peptidase domain (Peptidase_S9, Pfam 00326) belonging to Prolyl oligopeptidase family. An SSDB motif search indicates the possible presence of a peptidase domain in a couple of other family members but with low E value scores. Here, we have characterized one of these putative lysophospholipases (*P. falciparum* lysophospholipase, *Pf*LPL1; Gene ID: PF3D7_1476700). P*f*LPL1 is a protein of 353 amino acids that harbors a central lysophospholipase domain (27-111 aa; E value 3.97 e-10) and a Peptidase_S9 domain (265-352aa; 3.29 e-6) (Table S1). Homologues of P*f*LPL1 are also identified from P. berghei (PBANKA_122030), P. chabudi chabudi (PCHAS_13706), P. vivax strain Sal-1(PVX_096960), P. knowlesi (PKH_010790) and P. cynomolgi (PCYB _104230) using the genome database. An alignment of the predicted proteins sequences of these genes showed that *Pf*LPL1 is highly conserved among these *Plasmodium* species (Figure S1). A structural signature of lipase enzyme is the catalytic triad which is important for its catalytic activity. Sequence alignment showed presences of conserved Serine-167, Aspartate-300, and Histidine-330 residues, which are known to be involved in the formation of the catalytic triad. The nucleophilic serine residue of the active centre is present in a penta-peptide sequence GYSMG which is similar to the classic lipase motif GXSXG of α/β hydrolase (Figures S1and S2). To characterize the biochemical activity of P*f*LPL1, the recombinant protein corresponding to the full-length p*f*LPL1 gene was expressed in E. coli and purified by two-step affinity chromatography using Ni^2+^ NTA and amylose resins (Figure S3 A & B). The purified recombinant P*f*LPL1 was assessed for its lysophospholipase activity using an in vitro lysophospholipase assay. As shown in figure S3 C, the recombinant P*f*LPL1 showed concentration dependent lysophospholipase activity, Km and Vmax were determined using the Graph Pad Prism software (Km = 61.2 ±5.9 µM, Vmax= 2116.67 µM/mg/min) (Figure S3D).

### Endogenous tagging of *PfLPL1* gene and localization of *Pf*LPL1-GFP fusion protein in transgenic parasites

**Fig. 1:**
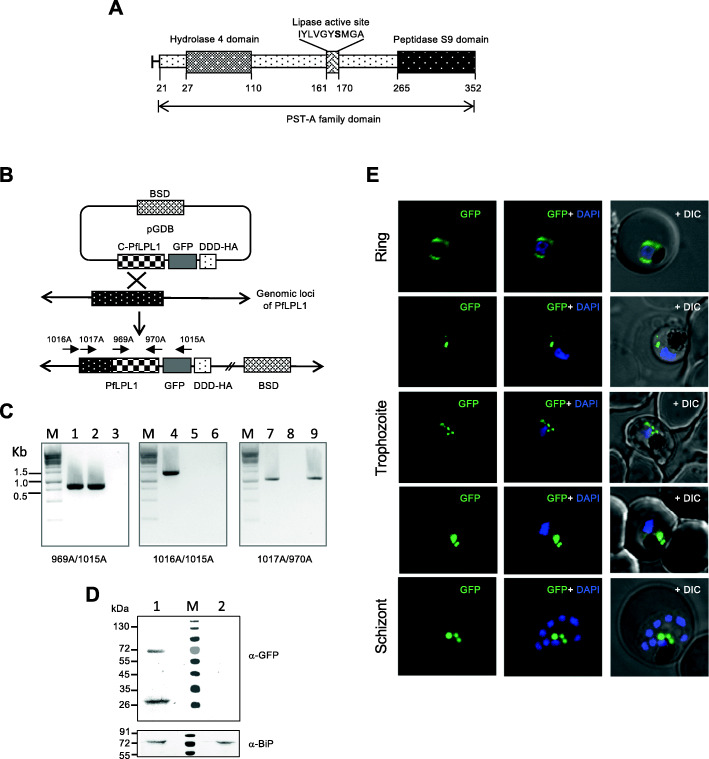
Generation of transgenic parasite expressing RFA-tagged (DDD-GFP) *Pf*LPL1 and localisation of *Pf*LPL1-GFP-DDD fusion protein in transgenic parasites. **a** Schematic representation of *Pf*LPL1 showing the hydrolase domain and putative peptidase domain. Locations of conserved residues of putative lipase/serine-hydrolase active site are also marked. This fixed pattern GXSXG including the active serine residue is conserved throughout all putative serine hydrolases. **b** Schematic diagram showing the strategy used to incorporate the regulatable fluorescent affinity (RFA) tag at the 3′ end of endogenous locus of *pfLPL1* through single cross over homologous recombination. The pGDB vector contains a blasticidin resistance cassette (BSD) for positive selection. The C-terminal fragment of *pfLPL1* gene was cloned in frame with GFP-DDD-tag which consists of *E. coli* dihydrofolate reductase degradation domain (DDD) with GFP and HA sequences. Schematic diagrams of the *pfLPL1*genomic loci before and after the homologous recombination are shown. Transgenic parasites obtained by selection over blasticidin, followed by on/off drug cycling to promote integration of the plasmid. Locations of PCR-primers are indicated in the schematic diagram. **c** PCR based analysis of transgenic parasite cultures to show integration of GFP-DDD-tag in the endogenous loci after drug cycling. Primers combinations are indicated for each panel: 969A/1015A (panel 1); 1016A/1015A (panel 2); 1017A/970A (panel 3); lanes 1, 4, and 7 are for transgenic parasite with integrated plasmid; lanes 2, 5, and 8 are for LPL-pGDB plasmid construct; lanes 3, 6, and 9 are for wild-type 3D7 parasites. **d** Immunoblot analysis using anti-GFP antibodies and trophozoite stage transgenic parasites expressing *Pf*LPL1-GFP-DDD A band of ~ 70 kDa, representing the fusion protein, and another band of ~ 26 kDa, representing GFP, are detected in the transgenic parasites (lane 2), but not in the wild-type parasites (lane 1). Lower panel shows a immunoblot for the same samples which was probed with anti-BiP antibodies; the BiP was detected in both the transgenic parasite lysate as well as wild-type parasite lysate. **e** Localization of *Pf*LPL1-GFP-DDD fusion protein in transgenic *P. falciparum* parasite cultures synchronized and visualized at different developmental stages. Fluorescent microscopic images of live transgenic parasites at ring (16–18 hpi), trophozoite (30 hpi), and schizont (42 hpi) stages. The parasite nuclei were stained with DAPI; fluorescence as well as DIC (differential interference contrast) images were obtained by confocal laser scanning microscope. NIS element software (version 4.0) was used to merge images obtained from different fluorescence/DIC images to produce the merged images. In ring stages, the GFP-fluorescence was observed around the nucleus; in early trophozoite stages, small vesicles are present near the parasitophorous vacuole and in the parasite cytosol. In mature stages, late trophozoite stage, and schizonts, GFP-fluorescence was observed in large vesicular structure in close association with the food-vacuole

To study the expression, localization, and essentiality of the *Pf*LPL1 in the parasite, we used the regulated fluorescent affinity (RFA) tag system for C-terminal tagging of the native gene [21]; the RFA tag includes GFP in frame with the *E. coli* degradation domain (DDD). Briefly, the parasites were grown in vitro on erythrocytes and transfected with the plasmid construct pGDB-*Pf*LPL1 (see the “Methods” section for details). The endogenous *pfLPL1* gene was tagged with GFP-DDD-tag by single-crossover homologous recombination of the plasmid with the main genome of the parasite (Fig. 1a–c), so that the expression of fusion protein was under the control of native promoter. The transgenic parasites showed expression of *Pf*LPL1-GFP-DDD fusion protein of ~ 70 kDa, migrating at the expected predicted size for the endogenously DDD-GFP tagged protein. Such a band could not be detected in wild-type 3D7 parasites (Fig. 1d).

**Fig. 2:**
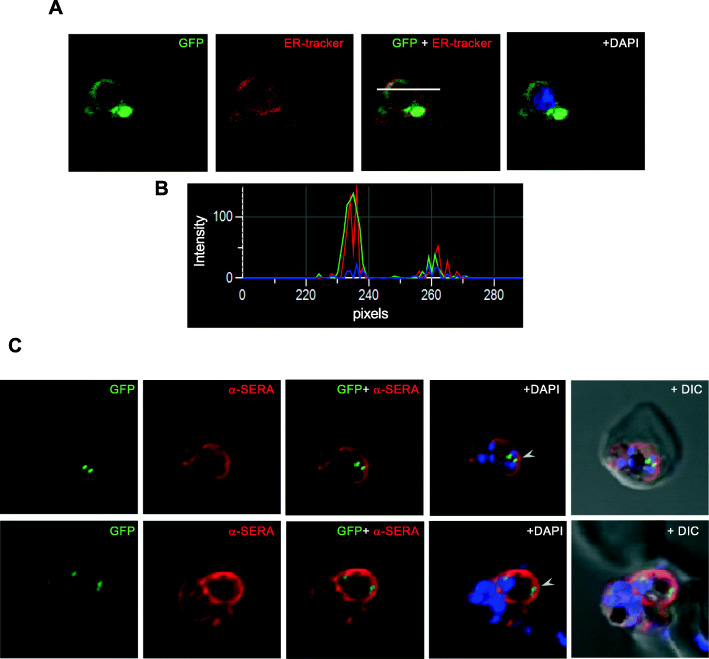
Sub-cellular localization of *Pf*LPL1-GFP-DDD fusion protein in transgenic parasite. **a** Fluorescent microscopic images of live transgenic parasites at ring stages stained with ER-Tracker (red). The parasite nuclei were stained with DAPI (blue), and slides were visualized by confocal laser scanning microscope. **b** A histogram plot for the fluorescence intensity analysis for GFP (green) and ER-tracker (red); the image was analysed by NIS elements software across the stained parasite (marked with white line). **c** Fluorescence images of transgenic parasites at trophozoite stages immuno-stained with antibodies of parasitophorous vacuole resident protein SERA (Serine Repeat Antigen) (red). The *Pf*LPL1-GFP-labelled vesicles are seen close to the parasite boundaries inside the parasite cytosol (marked with arrowhead). Parasite nuclei were stained with DAPI (blue) and the parasite were visualised by confocal laser scanning microscope

These transgenic parasites were studied for localization of the *Pf*LPL1-GFP-DDD fusion protein by confocal microscopy. In early stages of the parasite, rings, and young trophozoites, fluorescence of the GFP fusion protein was localized around the nucleus, which corresponds to the ER region (Figs. 1e, 2a). Indeed, staining by ER-tracker showed overlap with the GFP fluorescence in these parasites (Fig. 2a), as also confirmed by overlapping histogram plot for the fluorescence intensity of GFP and ER-tracker (Fig. 2b). In trophozoite stages, the GFP fluorescence was observed in distinct foci/vesicle-like structures in the parasite. In mid-trophozoite stages, these vesicles were observed near the parasite periphery towards parasitophorous vacuole (Fig. 1e). In later stages, a number of vesicles were observed further in the parasite cytosol and in close proximity of the food vacuole. In late-trophozoites and schizont stages, the GFP fluorescence accumulated near the food vacuole in 1 or 2 large structures (Fig. 1e, Figure S4A and B).

### *Pf*LPL1 containing vesicles traffic from parasite periphery towards vicinity of food-vacuole to develop into a large vesicular structure in asexual parasite stages

**Fig. 3:**
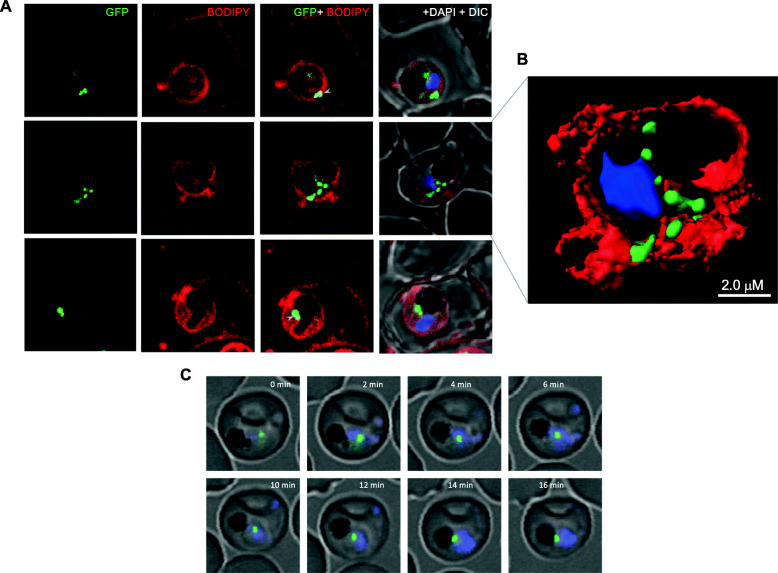
*Pf*LPL1 resides in vesicles which associate with parasite membrane and with food-vacuole. **a** Fluorescence images showing labelling of membranes in *P. falciparum* infected RBCs and localization of *Pf*LPL1. Trophozoite stage transgenic parasites expressing *Pf*LPL1-GFP-DDD were stained with BODIPY-TR ceramide (red); the parasite nuclei were stained with DAPI (blue) and visualized by confocal laser scanning microscope. Small GFP foci of the *Pf*LPL1-GFP-DDD fusion protein was observed near parasite boundary (panel 1, marked with arrowhead); these foci showed closed association with fluorescence by BODIPY-TR labelled parasite membrane. In some parasites, the GFP vesicles are seen in parasite cytosol (panel 2) and in close association with food-vacuole (panels 2 and 3 marked with arrowhead). **b** A three-dimensional reconstruction of series of Z-stack images (corresponding to panel 2 in **a**) using IMARIS software. Small GFP vesicles are present juxtaposed to the parasite membrane, in the parasite cytosol and close to the food-vacuole. **c** Time-lapse microscopy of *Pf*LPL1-GFP-DDD expressing transgenic parasites showing localization and migration GFP-labelled vesicles. Sequential images of a trophozoite stage parasite over a time interval of 16 min showing trafficking of a GFP-labelled vesicle in the parasite cytosol which subsequently gets associated at the boundary of food-vacuole (having dark hemozoin)

To further investigate these intriguing and dynamic localization patterns, a series of cellular staining and immuno-staining were carried out. The transgenic parasites were immuno-stained with anti-SERA, as a marker of the parasitophorous vacuole [24]. The anti-SERA antibody showed clear staining around parasite periphery defining the parasitophorous vacuole. The *Pf*LPL1-GFP-labelled vesicles were seen in close proximity with anti-SERA staining but did not overlapped with it, suggesting that the GFP-vesicles are present at parasite periphery within the parasite and turn towards the parasite cytosol (Fig. 2c). Further, we labelled the parasite membranes by a non-specific lipid membrane probe BODIPY-TR ceramide, which labels parasite plasma membrane, parasitophorous-vacuole membrane, tubulo-vesicular membranes, and food vacuole membrane. The *Pf*LPL1-GFP-labelled vesicles were observed in association with parasite boundary but clearly distinct from membrane labelling (Fig. 3a, Figure S5). A three-dimensional reconstruction based upon Z-stack images of these parasites confirmed close proximity (without direct association) of these vesicles with the parasite membrane (Fig. 3b). Importantly, initial observations suggested that the *Pf*LPL1-GFP is taken up by single membrane bound endocytic-like vesicles; these vesicles traffic through the parasite cytosol and then accumulated in close vicinity to the food vacuole (Fig. 3a, b and S5).

To further understand the trafficking of *Pf*LPL1 in the parasites, we determined the localization and movement of *Pf*LPL1-GFP-tagged vesicles in transgenic parasites by time-lapse confocal microscopy. Observations of trophozoite stage parasites expressing *Pf*LPL1-GFP using time-lapse imaging showed different phases of vesicles development and movement in the parasites (Fig. 3c). In this set of live-imaging, *Pf*LPL1-GFP containing endocytic vesicle like structure appeared near the parasite plasma membrane and then traverse through the parasite cytosol (0-4 min); this vesicle is present as individual GFP-labelled structure, which gradually migrates towards the food vacuole (6–14 min) and subsequently localizes in close proximity to the food-vacuole (Fig. 3c). In another set of images, a number of GFP-labelled vesicles are present near the parasite membrane. These vesicles subsequently migrated in the parasite cytosol and ultimately culminated as a single structure near the food-vacuole (Figure S4B). Overall, these results show migration of *Pf*LPL1-GFP containing vesicles in the parasite cytosol and their accumulation into a large vesicular body near the food-vacuole.

**Fig. 4:**
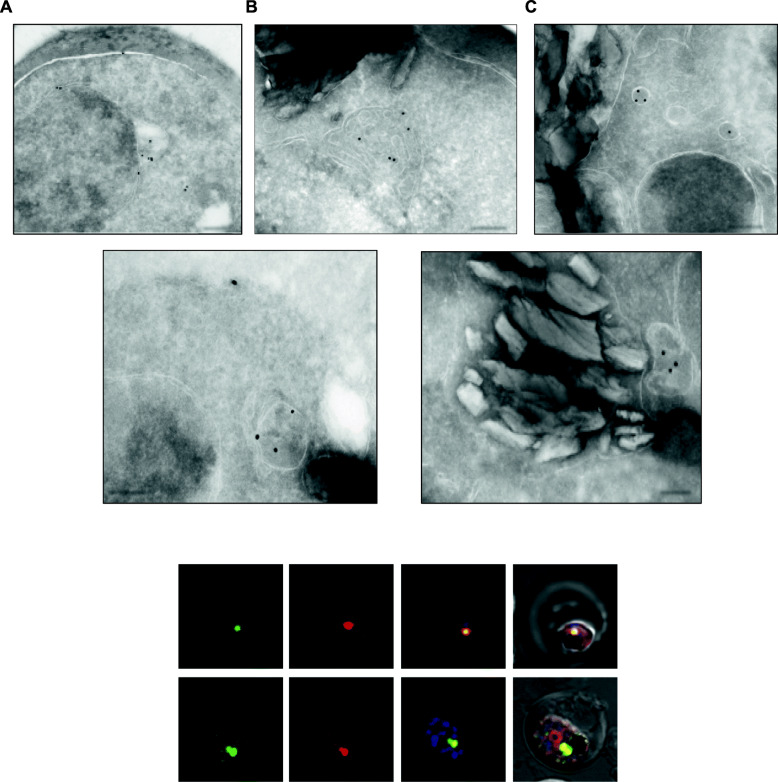
**a**–**e** Localization of *Pf*LPL1 by immune-electron microscopy. Ultra-thin sections of transgenic *P. falciparum* parasites expressing *Pf*LPL1-GFP-DDD were labelled with anti-GFP antibody and gold labelled secondary antibody: **a** in young parasites, labelling was observed around nucleus in the endoplasmic reticulum; **b** in some young parasite, labelling was in the putative ER exit site; **c** in trophozoite stages, labelling was observed in small vesicles (< 100 nm) in the parasite cytosol; **d**, **e** in mature parasites, labelling was observed in large vesicular structure (> 200 nm) localized in close association with the food-vacuole (having characteristic hemozoin). Scale bar = 200 nm. **f** *Pf*LPL1 associate with neutral lipid storage body near food vacuole. Fluorescence images of trophozoites stage transgenic parasites expressing *Pf*LPL1-GFP-DDD stained with Nile Red, a neutral lipid staining dye. The large GFP-labelled structure close to the food vacuole (having dark hemozoin) showed staining with Nile Red. The parasite nuclei were stained with DAPI (blue) and parasites were visualized by confocal laser scanning microscope

To further ascertain the association of *Pf*LPL1 with vesicular structures, we performed immuno-electron microscopic studies of the transgenic parasite using anti-GFP antibodies. In early ring stages, immuno-labelling of *Pf*LPL1-GFP was observed in the endo-membrane system of the parasite (nuclear envelope-ER region) (Fig. 4a, b). In trophozoite stages, the immuno-labelling was observed in small membrane bound vesicles of ~ 100 nm size, near the parasite plasma membrane (Fig. 4c); in addition, some vesicles were observed distributed in the parasite cytosol (Fig. 4c). Mature trophozoite stages displayed large, clearly labelled membrane bound structures (~ 200–300 nm), which were closely associated with the food-vacuole (Fig. 4d, e). Specificity of the observed structures for *Pf*LPL1-GFP vesicles was confirmed with immuno-EM controls conducted with secondary antibodies alone or pre-immune mice sera.

Overall localization analyses strongly indicate that (1) *Pf*LPL1 is associated with ER in early ring stages; (2) it gets further trafficked from ER towards parasite periphery during early trophozoite stage and gets associated with endosomal-like vesicles, which are developing during uptake of content of parasitophorous vacuole/host cytosol; (3) the *Pf*LPL1 protein subsequently traverses along with these endosomal like vesicles in parasite cytosol; and (4) these vesicles get associated/fuse with each other in the form a large vesicular structure near parasite food vacuole in mature parasite stages.

### *Pf*LPL1 associates with neutral lipid storage site (lipid bodies) in the parasite

Since *Pf*LPL1 is expected to generate free fatty acid (FA) from lyso-lipids, we assessed any potential association of the protein with neutral lipid bodies or lipid storage that could accumulate such FA. We determined localization of *Pf*LPL1 with respect to lipid storage vesicles using Nile Red. Nile Red is a hydrophobic probe that can stain lipid storages in cells and concentrate in stores of neutral lipids such as TAG [25]. The *Pf*LPL1-GFP-DDD parasites were stained with Nile Red and visualized with confocal microscopy. In trophozoite stage parasites, the Nile Red staining showed a single intensely fluorescent spot closely associated with the food vacuole (Fig. 4f). Similarly, 1–2 of these intensely labelled spots representing neutral lipid rich structures were observed in late schizont stages. The Nile Red stained lipid bodies showed complete overlap with GFP fluorescence near the food vacuole, suggesting that the large vesicular structures that contain *Pf*LPL1 near the parasite food vacuole are neutral lipid storage bodies (Fig. 4f).

To ascertain that the GFP-tag is not influencing the localization of the native protein in the parasite, we generated a transgenic parasite where the *pfLPL1* gene was tagged at the C-terminal to express the native gene fused with the HA-DD tag (Figure S6). Localization of the fusion protein was assessed by immuno-staining with anti-HA antibodies and Nile red staining. All the results obtained with *Pf*LPL1-GFP fusion parasite lines were confirmed in the *Pf*LPL1-HA-DD line: *Pf*LPL1 protein was localized in vesicular structures; these vesicles were also observed near the parasite periphery in early parasite stages, and in later stages, a number of vesicles were seen in the parasite cytosol and in close proximity of the food vacuole (Figure S7). In late-trophozoites and schizont stages, the protein was found to be accumulating near the food vacuole in 1 or 2 large vesicular structures (Figure S7A, B). These structures showed clear overlap with Nile Red staining in the parasites (Figure S7B).

Overall, localization studies by GFP-tagging/HA-tagging and Nile Red staining showed that the *Pf*LPL1 is associated with neutral lipid storages and thus likely to be involved in generation of neutral lipids in the parasite.

### Selective degradation of *Pf*LPL1 inhibits parasite growth and disrupts the intra-erythrocytic developmental cycle

**Fig. 5:**
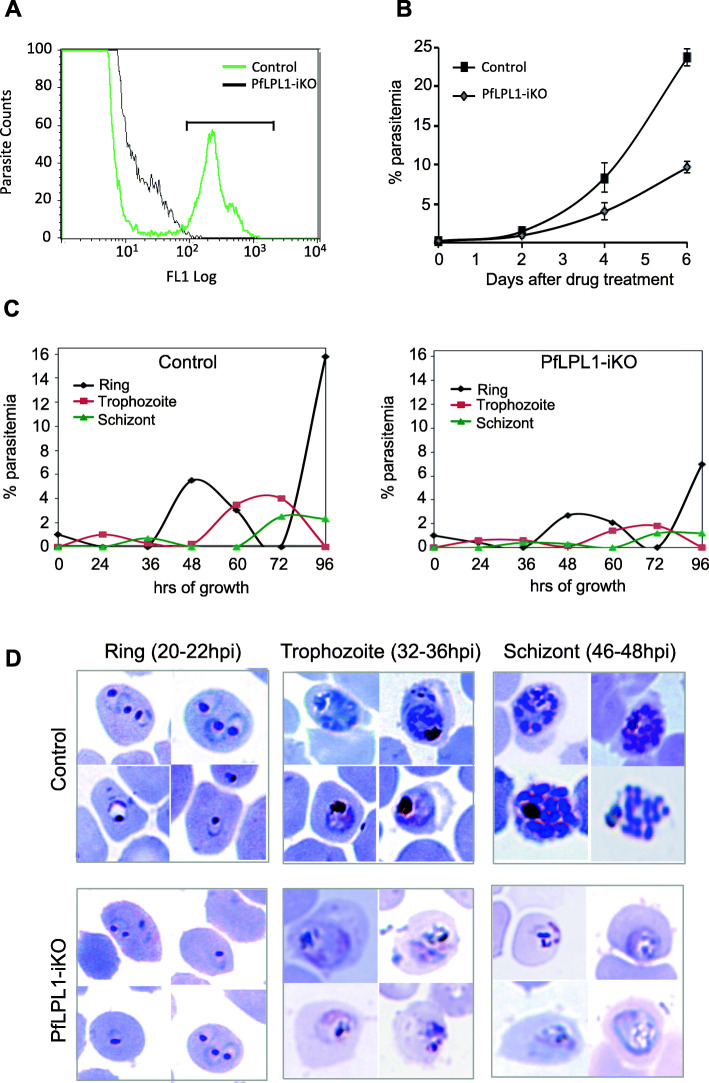
Inducible knock-down of *Pf*LPL1 protein in the transgenic parasites and its effect on growth and development asexual cycle of the parasites. Tightly synchronized ring stage parasite culture (0.2% parasitaemia) of transgenic parasites was grown with or without TMP (control and *Pf*LPL1 inducible knock-down, *Pf*LPL1-iKO, respectively). **a** Flow cytometry histogram showing reduction in population of GFP-labelled parasites in transgenic parasite cultures after *Pf*LPL1 inducible knock-down (grown in absence of TMP) as compared to control parasites (grown in presence of TMP). **b** Graph showing parasite growth for three asexual cell cycles of the parasite, as determined by counting total parasitaemia at 48, 96, and 144 h. All analyses were performed in triplicate or more (*n* = 3); the error bars indicate standard deviations. **c** Graphs showing parasite stage composition at different time points (0–120 h) in parasite culture from control and *Pf*LPL1-iKO sets. **d** Giemsa-stained images of parasites showing effect on parasite morphology at different time points (0–48 h) in parasite culture control and *Pf*LPL1-iKO sets

To understand the functional significance of *Pf*LPL1 and its possible involvement in neutral lipid synthesis/storage, we utilized the DDD-tag mediated selective degradation of *Pf*LPL1 in the transgenic parasites. Selective degradation of GFP-DDD-tagged protein in absence of TMP was assessed by quantifying fluorescent cells in parasite cultures, which were grown in presence or absence of TMP. As shown in Fig. 5a, absence of TMP caused significant reduction in fluorescent intensity of parasites and reduced ~ 90% of fluorescent parasite population as estimated by flow-cytometry based analysis. Effect of this inducible knock-out of *Pf*LPL1 (*Pf*LPL1-iKO) was assessed on parasite growth in vitro by estimating development of new ring stage parasites for 3 cycles. In *Pf*LPL1-iKO set, the parasite growth was significantly reduced (~ 60%) as compared to control parasite culture (Fig. 5b). Growth inhibition studies using *Pf*LPL1-HA-DD lines showed much enhanced effect; inducible knock-down of *Pf*LPL1 in iKO set showed ~ 80% growth inhibition as compared to control set (Figure S8). These results suggest that *Pf*LPL1 plays a critical role for the intra-erythrocytic development of *P. falciparum*.

To study the effect of selective degradation of *Pf*LPL1 on parasite development and morphology, synchronized ring stage parasites in *Pf*LPL1-iKO and control sets were analysed for several intra-erythrocytic cycles. In the control set, during each intra-erythrocytic cycle, the parasites developed from rings to trophozoites to mature schizonts and subsequently into merozoites, which were released from the schizonts invaded new erythrocytes, which effectively increased the total parasitaemia about 6 times (Figs. 5c, d, S9). However, in the *Pf*LPL1-iKO set, development of the parasite from trophozoites to schizont was hindered (Fig. 5c, d). The *Pf*LPL1-iKO set also showed morphological abnormalities during parasite development. The mature trophozoite stages showed abnormal food-vacuoles without distinct hemozoin, as compared to control parasites that showed clear dark hemozoin pigment (Fig. 5d). This resulted in reduction in parasitaemia in the next cell cycle. Reduction in the parasitaemia was also detected in subsequent cycles leading to a significant reduction in overall growth of the parasites.

### Knock-down of *Pf*LPL1 resulted in reduction of food-vacuole associated neutral lipids

**Fig. 6:**
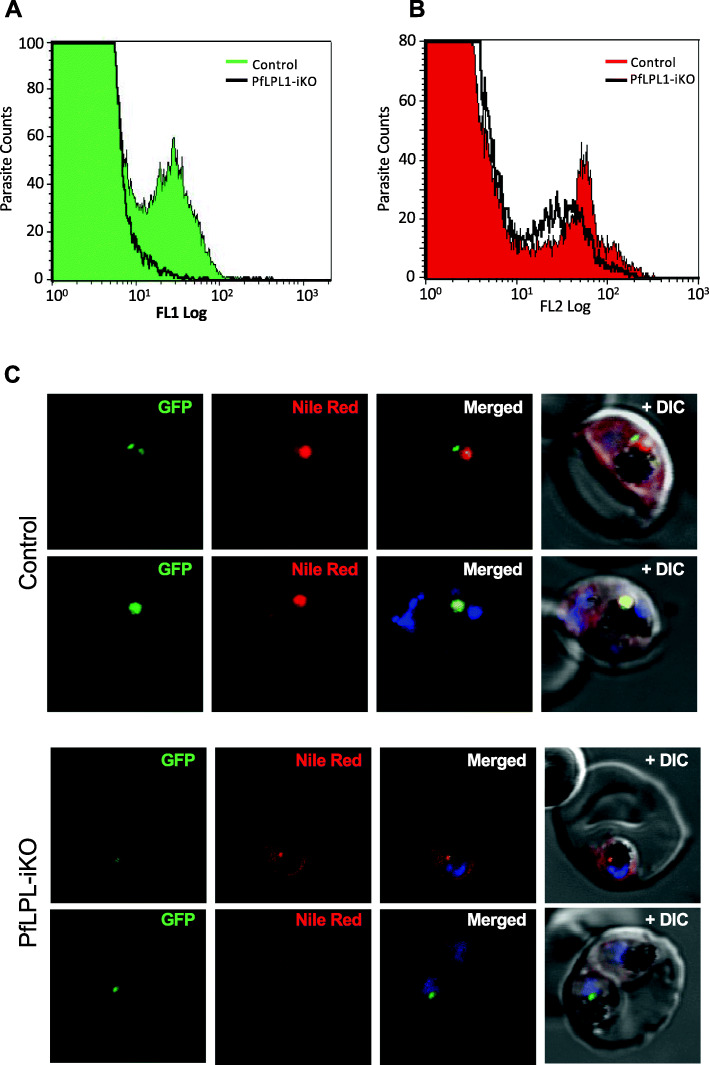
Inducible knock-down of *Pf*LPL1 protein in the transgenic parasites and its effect on the development of neutral lipid body. **a**, **b** Synchronous transgenic parasites at ring stages were grown till late trophozoite stages in control and *Pf*LPL1-iKO sets, stained with Nile red and analysed by flow cytometry. Flow cytometry histogram showing concomitant reduction in GFP fluorescence (FL-1) and Nile red labelling (FL-2) in parasites after *Pf*LPL1-iKO as compared to control parasites. **c** Fluorescence images of trophozoites stage transgenic parasites in *Pf*LPL1-iKO set, showing the reduction in Nile red fluorescence intensity and loss of GFP-fluorescence as compared to control parasite. The parasite nuclei were stained with DAPI (blue) and parasites were visualized by confocal laser scanning microscope

To understand the possible role of *Pf*LPL1 in biogenesis and storage of neutral lipids, we assessed any effect of selective knock-down of *Pf*LPL1 levels in the parasite on the accumulation of neutral lipids. Synchronized ring stage *Pf*LPL1-GFP-DDD parasites in control and *Pf*LPL1-iKO sets were grown for 24–30 h to develop into trophozoite stages. Subsequently, levels of *Pf*LPL1-GFP and neutral lipids levels were assessed in these parasites. Flow cytometry-based analysis as well as fluorescence microscopy showed a significant reduction of GFP fluorescence in parasite population (Figs. 6a, c and S10) as well as reduction in Nile Red staining of lipid storage bodies (Figs. 6b, c and S10) after downregulation of *Pf*LPL1 (*Pf*LPL1-iKO), as compared to control parasites. Inducible knock-down studies using *Pf*LPL1-HA-DD lines showed a similar effect on reduction in neutral lipid storage body (Figure S8C).

### Downregulation of *Pf*LPL1 results in major changes in the parasite lipid composition and reveals its potential role for TAG synthesis

**Fig. 7:**
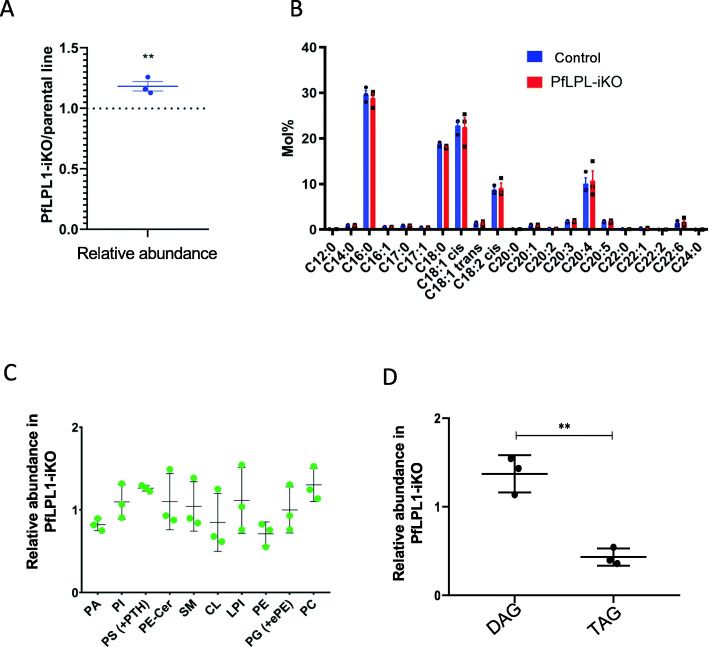
Inducible knock-down of *Pf*LPL1 protein in the transgenic parasites and its effect on lipid homeostasis. Synchronous transgenic parasites at ring stages were grown till late trophozoite stages and lipids compositions were assessed by mass spectrometry-based lipidomic analyses from *Pf*LPL1-iKO and control sets. **a** Analysis of relative amount of total fatty -acids (reflecting the FA composition of existing phospholipids + neutral lipids + free FA pool) in *Pf*LPL1-iKO as compared to control set. **b** Analysis of fatty acids (FAs) showing no significant changes in the FA composition of parasites from control and *Pf*LPL1-iKO sets. **c** Analysis of the parasites phospholipid composition showing downregulation of phosphatidic acid (PA) and phsophatidylethanolamine (PE) and upregulation of phosphatidylcholine (PC) and phosphatidylserine (PS) level in the parasite after knock-down of *Pf*LPL1. **d** Analysis of major neutral lipids levels showing upregulation of diacylglycerols (DAGs) and downregulation of triacylglycerols (TAGs) in *Pf*LPL1-iKO set. All analyses were performed in triplicate (*n* = 3) or more; the error bars indicate standard deviations. The *p* values were calculated by Student’s *t* test: *p < 0.01, **p < 0.005, and ***p < 0.001. PA, phosphatidic acid; PE, phsophatidylethanolamine; PC, phosphatidylcholine; PS, phosphatidylserine; PI, phosphatidylinositol; SM, sphingomyelin; CL, cardiolipin; LPI, lysophosphatidylinositol; and PG, phosphatidylglycerol

To determine the role of *Pf*LPL1 at the lipid synthesis levels and its putative associated function with neutral lipid storage, we conducted mass spectrometry-based lipidomic analyses on lipid extracted from synchronized *Pf*LPL1-GFP-DDD ring stage parasites grown further for 24 h from control as well as from *Pf*LPL1-iKO sets. We first analysed the FA content of the mutant by GCMS approach. This revealed that the disruption of *Pf*LPL1 induced an increase in total FA content. The total FA content reflects an increase of FA from existing phospholipid-neutral lipids and free FA pools. Detailed analysis of FA composition showed no significant changes in the FA molecular species of parasites from control and *Pf*LPL1-iKO (Fig. 7a), where the main FA species are C16:0, C18:0, and C18:1 as previously reported [6, 26]. To determine which lipid class was specifically affected in the *Pf*LPL1-iKO, we further analysed the phospholipid and neutral lipid content. The phospholipid composition revealed significant changes in *Pf*LPL1-iKO set. Indeed, relative abundance of phosphatidic acid (PA) and phosphatidylethanolamine (PE) were significantly reduced in the knock-down culture, whereas relative abundance of both phosphatidylcholine (PC) and phosphatidylserine (PS) were significantly increased (Fig. 7b). Importantly, analysis of the content of major neutral lipids making the bulk of lipid bodies composition, i.e. diacylglycerol (DAG) and triacylglycerol (TAG), showed that the relative abundance of DAG was significantly increased, whereas the one from TAG was significantly reduced after *Pf*LPL1 knock-down (Fig. 7c). Together, these results correlate with our phenotypic analysis that *Pf*LPL1 is involved in the generation of neutral lipids, and more particularly TAGs, in addition it also impacts the content of the parasite phospholipid.

### Reduction in *Pf*LPL1 levels disrupts hemozoin formation in the parasite

**Fig. 8:**
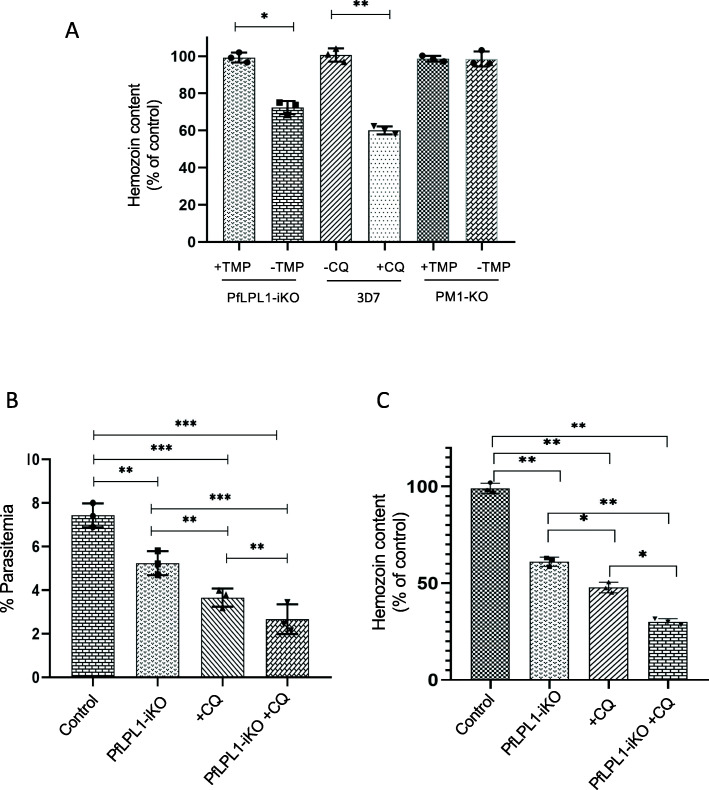
Downregulation of *Pf*LPL1 protein in transgenic parasites and its effect on the formation of hemozoin in the food vacuole. Synchronous transgenic parasites at ring stages were grown till late trophozoite stages (*Pf*LPL1-iKO and control sets), and parasite growth as well as total hemozoin content was assessed as compared to control. **a** Graph showing reduction in hemozoin content in transgenic parasites *Pf*LPL1-iKO (-TMP) as compared to control parasites (+TMP). The parent PM1-KO parasite line was used as control. The 3D7 parasite line treated with chloroquine was used as positive control showing significant reduction as compared to untreated controls. **b** The graph showing effect of chloroquine treatment on parasite growth in *Pf*LPL1-iKO set as compared to control and chloroquine only set. **c** The graph showing effect of chloroquine treatment on reduction in hemozoin content in *Pf*LPL1-iKO set as compared to control and chloroquine only set. The chloroquine showed additive effect for inhibition of parasite growth as well as hemozoin formation. The *p* values were calculated by Student’s *t* test: *p < 0.01, **p < 0.005, and ***p < 0.001

The localization pattern, knockdown, and lipidomic analysis clearly suggested involvement of *Pf*LPL1 in generation/storage of the neutral lipids in the parasite; neutral lipids are known to be efficient catalysts of hemozoin formation in the parasite [10, 11, 27–29]. Our data also showed that downregulation of *Pf*LPL1 caused food-vacuole abnormalities associated with hemozoin development in the parasite. To ascertain the relation between hemozoin development and reduction in neutral lipids after downregulation of *Pf*LPL1, we assessed total hemozoin content after selective knockdown of *Pf*LPL1 in the transgenic parasites. Total hemozoin content was estimated in equal number of trophozoite stage parasites grown in control (with TMP) and *Pf*LPL1-iKO (without TMP) sets. The transgenic parasites growing in the *Pf*LPL1-iKO set showed ~ 40% reduction in hemozoin levels as compared to the control set. The wild-type *P. falciparum* 3D7 parasite culture treated with chloroquine, which is known to interfere with hemozoin formation [30, 31], was used as a positive control, these parasites showed ~ 45% reduction in hemozoin levels (Fig. 8a). The control parasite line PM1KO showed no difference in hemozoin levels when grown in presence or absence of TMP (Fig. 8a). Further, we also assessed any synergistic/additive effect of chloroquine treatment with the inducible knock-down of *Pf*LPL1. The transgenic parasites were treated with chloroquine in both control and *Pf*LPL1-iKO sets. The chloroquine produced additive effect with *Pf*LPL1-iKO for parasite growth inhibition as well as for inhibition of the hemozoin formation (Fig. 8b, c). Together, this shows that the reduction of hemozoin is specific to the deletion of *Pf*LPL1.

### Host-derived FAs can complement *Pf*LPL1 iKO

**Fig 9:**
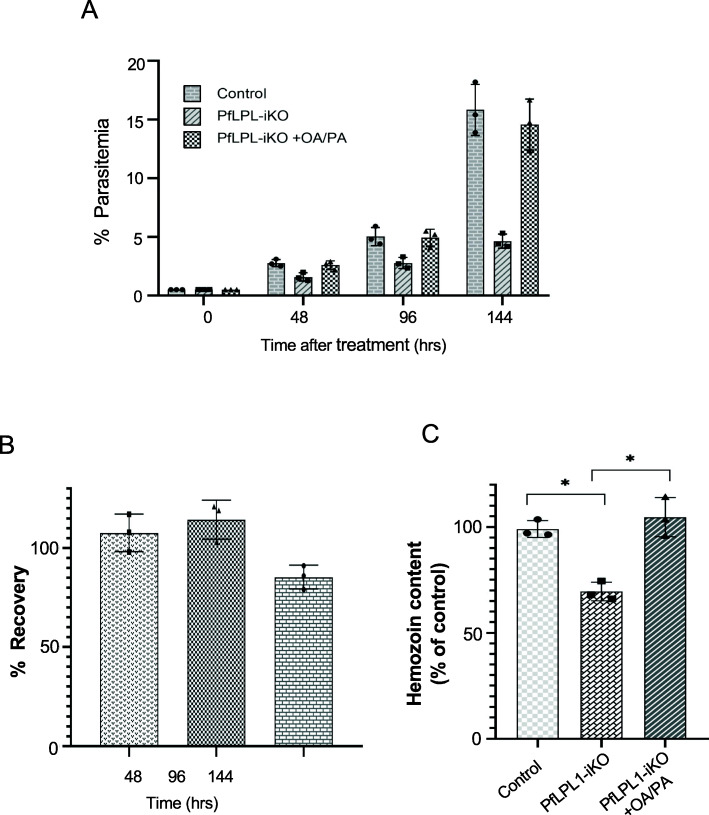
Host-derived FAs can complement *Pf*LPL1 iKO. Synchronous transgenic parasites in the *Pf*LPL1-iKO set in the presence or absence of FAs (oleic acid + palmitic acid); the parasite growth as well as total hemozoin content was assessed as compared to control set. **a**, **b** Graph showing % parasitaemia and % recovery in *Pf*LPL1-iKO + FAs set as compared to *Pf*LPL1-iKO at different time points after treatment. **c** Graph showing hemozoin content in *Pf*LPL1-iKO + FAs set as compared to *Pf*LPL1-iKO

We further confirmed the role of *Pf*LPL1 in generating FAs, required for neutral lipid development, which are ultimately used in hemozoin formation, by complementation assay. The *Pf*LPL1-iKO set was complemented with two FA species which are known to be essential for blood stages, namely C16:0 and C18:1 (palmitic acid and oleic acid, respectively) [32, 33]. The complementation effectively restored 80–100% parasite growth (Fig. 9a, b) at different time points during parasite growth (at 48 h, 96 h, and 144 h post invasion). In addition, and more importantly, the complementation fully restored the level of hemozoin in the *Pf*LPL1-iKO set as compared to control (Fig. 9c). Further, we also complemented the *Pf*LPL1-iKO set with excess LPC in the culture media. However, there was no complementation in parasite growth or hemozoin formation in the iKO set (Figure S11).

## Discussion

The establishment of the intra-erythrocytic *P. falciparum* infection is associated with a large increase in the phospholipid, neutral lipid, and lipid-associated fatty acid (FA) content in infected red blood cells [12]. In addition to de novo synthesis, scavenging of lipids/phospholipids from the host milieu and their catabolism may play a crucial role to fulfil the parasite requirement of lipids during the erythrocytic cycle. In our attempts to further understand the process of catabolism of phospholipids in the parasite, we characterized a putative lysophospholipase in *P. falciparum*, *Pf*LPL1, which is a member of the family of lysophospholipase-like protein in the parasite. Biochemical characterization of recombinant *Pf*LPL1 showed that it harbours lysophospholipase activity (See supplementary data, Figure S3). Novel tools have been developed for transient down regulation of proteins in *Plasmodium* by tagging native genes with degradation domain etc. [34–37]. Here, we have used two different regulated C-terminal tags for transient knock-down of *Pf*LPL1 in the transgenic parasites; in one strategy, *Pf*LPL1 was expressed as a fusion protein with GFP and *E. coli* DHFR degradation domain (GFP-DDD) [21], whereas in the second strategy, *Pf*LPL1 was expressed as a fusion protein with HA-tag and FKBP destabilization (HA-DD) [34, 38].

Confocal microscopic analysis using transgenic parasites expressing *Pf*LPL1-GFP-DDD fusion protein showed that the protein is associated with a vesicular system in the parasite. The parasite is known to uptake the content of host erythrocyte cytosol, especially the host haemoglobin, as a major source of amino acids. The haemoglobin is taken up through the cytostome into double membrane bound vesicles, cytostomal vesicles, which subsequently fuse with the food vacuole [39, 40]; during this process, the outer membrane of these vesicles gets fused with the food-vacuole membrane, whereas the inner membrane is suggested to get degraded by phospholipases [10]. Our study suggests that *Pf*LPL1 trafficked from ER to PV, and subsequently, it is taken up by single membrane bound endosomal-like vesicles possibly along with other content from PV. These *Pf*LPL1-associated vesicles were not found to fuse with the food-vacuole; the *Pf*LPL1 staining was also not seen inside the food-vacuole. Rather, the *Pf*LPL1-vesicles accumulated into a large vesicular structure in close proximity of the food-vacuole. Immuno-electron microscopic studies also showed that the *Pf*LPL1 is enclosed in the membrane bound vesicles near the parasite periphery as well as in the parasite cytosol. Indeed, a dynamin-like machinery is shown to be involved in the generation of endosomal-like vesicles from parasite boundary; it was shown that these vesicle traffic to the lipid storage body near food-vacuole [41]. Further, localization of fusion protein in large vesicular structures near the food-vacuole showed resemblance with the previously reported neutral lipid bodies, which are known to be intimately associated with the food vacuole [10]. These lipid bodies are the sites of storage of di- and tri-acylglycerols (DAGs and TAGs). These neutral lipids are possibly obtained by the parasite by scavenging or these are generated by digestion of phospholipids during membrane recycling [42, 43]. Co-staining of neutral lipid body (NLB) in the transgenic parasites confirmed association of *Pf*LPL1 with these lipid bodies. These results may suggest that the *Pf*LPL1 is involved in generation of neutral lipids from complex phospholipids. These phospholipids may have been taken up by the parasite from the host. The malaria parasite is known to take up exogenous lipids and lipid precursors during blood stage cycle [6, 26]. *P. falciparum* blood stages are also able to survive in a minimal medium, only containing two sources of fatty acids, C16:0 and C18:1, suggesting that host scavenging is essential for the parasite survival [44, 45]. Incorporation of exogenous lysophosphocholine is also shown to be necessary for growth of *Plasmodium* as building block for active membrane biogenesis and asexual division [46]. It has also been shown that *Plasmodium* infected erythrocytes are able to scavenge substantial amount of albumin bound lysophosphocholine from host plasma that is catabolized to generate free fatty acids that are used for de novo lipid synthesis in the parasite [47]. Furthermore, the apicoplast lipid composition seems to be largely depending on lipid scavenged from the host during blood stages as well [6]. The lysophosphatidylcholines (LPCs) and phosphatidylcholines (PCs), the major phospholipids detected in the human serums, could be taken up by the parasite [14]. It is also shown that phosphocholine can be readily transferred from the erythrocyte membrane to intraerythrocyte parasite suggesting presence of a selective machinery for phosphocholine transport from host cell to the parasite [48]. Indeed, a phospholipid transfer protein is identified in *P. falciparum* that can transport different phospholipids between the membranes; this transporter is exported into the erythrocyte cytosol, and it is closely associated with parasitophorous vacuole [49]. Although the exact role of this transporter is not clear, but it is essential for survival of the parasite [50]; it may be involved in the transport of phosphocholine and other phospholipids from the erythrocyte membrane to the parasitophorous vacuole. These phospholipids may be taken up by the parasite by endocytosis or along with the host cytosol uptake. Our data showed that the *Pf*LPL1 gets entrapped in small endocytic vesicles near parasite periphery and subsequently traffic towards neutral lipid stores, which indicate that the *Pf*LPL1 is involved in catabolism of phospholipids/lysophospholipids as these are scavenged from the host or taken up during the process of endocytosis. The action of *Pf*LPL1 on complex phospholipids, such as lysophosphocholine, can generate fatty acids and glycerophosphocholine, which can be subsequently catabolized by enzyme glycerolphosphodiestere phosphodiesterase (GDPD) to generate glycerol-3-phosphate and choline. The recombinant *Pf*LPL1 was able to carry out this reaction in vitro. Further, the GDPD has recently been identified and characterized in *P. falciparum* [51].

The transient downregulation of *Pf*LPL1 using RFA system [21] caused reduction in neutral lipid stores. This strongly indicates that *Pf*LPL1 is involved in production of neutral lipids in the parasites. TAGs are the major acylglycerol detected in the infected erythrocytes [12]. Further, the Nile Red staining studies suggested that these TAGs could be stored in the lipid storage body associated with the food-vacuole. The TAGs are synthesized through a three sequential acylation steps from a glycerol-3-phosphate backbone. Usually, first two steps are catalyzed by glycerol-3-phosphate acyltransferase (GPAT), producing LysoPA (LPA). GPAT are present in two copies of different origins in the parasite genome, one encoding a eukaryotic GPAT in the ER, *Pf*GatP, and seems essential for blood stages [52], whilst the other one is of plant origin and present in the apicoplast, *Pf*apiG3PAT or ATS1, and is rather essential in liver stages and in *T. gondii* where it generates LPA backbone for the bulk phospholipid synthesis [10, 52, 53]. The second step is catalyzed by acyl-glycerol-3-phosphate acyltransferase (AGPAT), which use LPA to add a second FA and form PA. AGPATs are also present in two copies of different origin (eukaryotic and possibly plant-like) and seem important for parasite survival but their exact role for lipid synthesis is yet to be determined. The PA formed by GPATs and AGPATs is then dephosphorylated by phosphatidic acid phosphatases (PAP) to form DAG. Putative PAPs have not been characterized yet and their function and role remain to be elucidated although the parasite genome seems to express 2–3 putative PAPs [53]. The final acyl transfer is carried out by the enzyme acyl CoA: DAG acyltransferase (DGAT), which transfer a third fatty acid onto the *sn*-3 position of the glycerol backbone from DAG. Previous work reported that *P. falciparum* encodes for a single homologue of DGAT that is highly expressed during late trophozoite and schizont stages and might be essential for parasite survival. This DGAT likely allows the de novo synthesis of TAG during *P. falciparum* blood stages [9, 43]. Glycerol-3-phosphte, DAG, and FA that are generated by *Pf*LPL1 and *Pf*GDPD could then be assembled via the action of GPATs and AGPATs and subsequently utilized for TAG biosynthesis. Therefore, lipid recycling in the parasite involves catabolism of phospholipids and synthesis of neutral lipids simultaneously.

Our lipidomic analysis revealed that there was no change in the FA composition of parasites lacking *Pf*LPL1, suggesting that the enzyme does not have a specific FA species substrate affinity, for which reduction(s) would have been quantified. Reduction of PA content in the mutant could indicate that the FA generated via the action of *Pf*LPL1 could fuel the ER resident acyltransferases GPAT and/or AGPAT putatively responsible for the PA de novo synthesis. PA is used as a precursor for PE synthesis via the Kennedy pathway [32, 54]; the reduced PA levels would thus impact PE synthesis. Similarly, PS requires the scavenging of serine, which is massively incorporated for the synthesis of PS via the CDP-DAG pathway. Its synthesis would be favoured against the Kennedy pathway due to the lack of its PA substrate. The PC increase might be linked to (i) putative polar head exchange between PS and PC as previously suggested [5] or (ii) the putative increase in LPC not being degraded into FA+glycero-3-phosphocholine and thus directly used to form PC by the action of acyltransferases and *Pf*PMT [14, 55] generating phosphocholine from the unused phosphoethanolamine that is normally used for PE synthesis. Importantly, our results clearly point at a significant reduction of TAGs upon *Pf*LPL1 disruption, correlating and confirming our cellular evidences for the role of this enzyme in TAG synthesis, likely by catabolism of scavenged host/external environment phospholipids. The concomitant increase of DAG upon *Pf*LPL1 disruption intuitively suggests that DAG constitutes the likely acceptors of FA provided by *Pf*LPL1 to form TAGs via the action of the sole DGAT of the parasite.

In vitro activity-based assay showed that *Pf*LPL1 can cleave LPC, a standard substrate of lysophospholipase. However, LPC could not be quantified in the lipidomic analysis; this is actually the case for another lipid present in minute amount and barely detectable by any approach, yet essential for many metabolic pathways: phosphatidic acid (PA), which has to be metabolized via many essential enzymes [8, 56, 57]. It is possible that *Pf*LPL1 generates fatty acids through hydrolysis of other complex (lyso)phospholipids as substrates, but it does not rule out LPC as the potential substrate. Further work to determine which is the direct scavenged substrate of *Pf*LPL1 would be interesting. Transient downregulation of *Pf*LPL1 caused growth retardation of transgenic parasites. Further, the parasite developmental-stage profile showed that the development of trophozoites into schizonts was severely disrupted in these parasites. Overall, these results showed that *Pf*LPL1 plays functionally important role in parasite growth and development; the *Pf*LPL1 is involved in catabolism of phospholipids which is needed for production of TAGs in the parasite; downregulation of *Pf*LPL1 and subsequent reduction in TAGs stores caused severe growth retardation in the asexual stage parasites.

The exact role of TAGs stores in the parasite is not very clear; however, our results and previous studies with lipid labelling dyes showed that there is active synthesis of TAGs in the asexual stage parasite [10]. It is suggested that TAGs stores may not be involved de novo synthesis of membrane lipids and membrane biogenesis [43]. Further, the parasite may not be able to use TAGs as substrates for oxidative catabolism as unlike *T. gondii* it lacks most enzymes involved in beta-oxidation of FA [57]. Our data shows that de novo synthesized TAGs in the parasite might play important role its growth and development. The close association of TAGs stores with the food-vacuole suggests that their role in parasite is associated with processes in the food-vacuole. The parasite food-vacuole is mainly involved in proteolytic degradation of host haemoglobin and sequestration of released heme in the form of hemozoin; the hemozoin formation pathways is the major target of known drugs and is also targeted to develop new anti-malarials [58]. Several lines of studies have suggested that neutral lipids might play important catalytic role in hemozoin formation; the neutral lipids form nanosphere provide unique environment that promote hemozoin development [11, 27–29]. Our studies showed that downregulation of *Pf*LPL1 and subsequent reduction in TAGs stores effected development of parasite at trophozoite stages with abnormal food-vacuole morphology without clearly developed hemozoin. The total hemozoin estimation data correlated with these observations, as there was clear reduction in hemozoin levels after *Pf*LPL1 knock-down in the parasites. It is possible that the reduction in hemozoin could be due to reduced uptake of haemoglobin; however, there are no reports of involvement of neutral lipids in haemoglobin uptake, whereas neutral lipids are known to play role in hemozoin formation. Thus, our data strongly suggest that *Pf*LPL1 has a role in hemozoin formation through the generation of neutral lipids. We acknowledge that there is a mismatch in the timing of *Pf*LPL1 localization in the neutral lipid body (NLB) with that of hemozoin formation in the parasite; the hemozoin formation begins in the ring stage and occurs quite strongly in the early to mid-trophozoite stage, whereas we detect the appearance of *Pf*LPL1 in the neutral lipid body (NLB) starting from the mid-trophozoite stage. However, this apparent mismatch in timing could be due to a low level of *Pf*LPL1expression, insufficient for detection in microscopy, but sufficient to produce the required lipids in the early stage of the parasite cycle.

This study shows that *Pf*LPL1 associates with the NLB, and this lipase degrades complex phospholipids to generate FAs required for neutral lipid synthesis. The lipid storage bodies are suggested to provide neutral lipid, whenever required by the cell for its development and synthesis of new membranes [59, 60]. Since the NLBs are closely associated with food-vacuole, it is hypothesized that these NLB provide neutral lipids to food vacuole as required; whereas *Pf*LPL1 knock-down clearly reduced the neutral lipid content as well as hemozoin development. These results suggest that neutral lipids stored in close association with the parasite food-vacuole are involved in hemozoin development. The complementation assay further confirmed this this hypothesis. Complementation of the *Pf*LPL1-iKO with C16:0 and C18:1 (palmitic acid and oleic acid, respectively), the only two FA species known to be essential for blood stage parasites, fully restored parasite growth as well as the level of hemozoin after *Pf*LPL1-iKO. However, there was no complementation in parasite growth as well as hemozoin formation with LPC. These results may suggest one or more of the following circumstances for the *Pf*LPL1-iKO parasite set: (i) since there was significant reduction in *Pf*LPL1 enzymes, the remaining LPL1 in the iKO set was not enough to rescue the growth and to hemozoin formation even in presence excess LPC; (ii) sufficient/excess amount of LPC was already present in the milieu and excess amount of substrate was not helpful to revive the enzymatic reaction with remaining *Pf*LPL1 protein; or (iii) the *Pf*LPL1 generates fatty acids through hydrolysis of some other complex phospholipids as substrate. Taken together, this set of data directly links the input of scavenged fatty acids with the role of *Pf*LPL1 in hemozoin formation and thus greatly improves our understanding of the function of *Pf*LPL1 and parasite biology.

## Conclusions

Overall, our data showed that the hemozoin development in the food-vacuole is dependent upon TAGs in the neutral-lipid body; these TAGs are generated through phospholipid catabolism by *Pf*LPL1 in the parasite. The metabolic pathways of phospholipid recycling and de novo lipid synthesis are therefore crucial for parasite survival. Detailed understanding of neutral lipid-based heme detoxification via TAG and inhibition of *Pf*LPL1 may help us to design efficient novel anti-malarial strategies.

## Methods

### Expression, purification of recombinant P*f*LPL1 and enzyme activity assay

A DNA fragment corresponding to full length p*f*LPL1 gene (1aa - 353 aa) containing the lipase domain was amplified by PCR using P. falciparum 3D7 genomic DNA. The amplified fragment was cloned in the NcoI and XhoI sites of pETM-41 expression vector. The resultant plasmid pETM-41-p*f*LPL1 was transformed into E. coli BL21(DE3) cells for expression of the recombinant protein. These E. coli BL21(DE3) cells were grown in Luria broth containing Ampicillin (100μg/ml) at 37°C under shaking to an OD600 of 0.6-0.7 and expression of recombinant protein was induced with isopropyl-β-D-1- thioglactopyranoside (IPTG) at a final concentration of 1 mM. The recombinant P*f*LPL1 was purified from cytosolic fractions of the E. coli cell lysate by two-step affinity chromatography using Ni^2+^ NTA and amylose resins.

The purified recombinant P*f*LPL1 was assessed for its lysophospholipase activity using an in vitro lysophospholipase assay modified from Kishimoto et.al [61] and Mohanty et. al [62] using Amplex Red Phospholipase kit (Thermo Fischer Scientific) as per manufacturer’s instructions. The reaction mechanism of the enzymatic assay involves two-step hydrolysis of LPC by lysophospholipase to glycerophosphoryl-choline (GPC), which is further hydrolysed to generate glycerophosphate and choline. The choline is further oxidized by choline oxidase to betaine and H_2_ O_2_. Finally, H_2_ O_2_, in the presence of horseradish peroxidase, reacts with Amplex Red reagent (10-acetyl-3,7-dihydrophenoxazine) to yield a highly fluorescent product, resorufin, fluorescence in the reaction mix is measured at 530/590 nm. The assay was performed in a 200 μl volume containing 10μg of recombinant protein, in 1X reaction buffer (250 mM Tris-HCl, 25mM CaCl_2_, pH 8.0), 20mM H_2_ O_2_, 100μM Amplex Red reagent, 2 U/mL HRP, 0.2 U/mL choline oxidase, 0.5mM lecithin and 0.1U GPCP. The enzyme activity is recorded as the increase in fluorescence (excitation 530 nm; emission 590 nm) for 3-6h at room-temperature using a LS50B Fluorometer (Perkin-Elmer). The standard curve was prepared using different concentration of resorufin and activity of recombinant protein was calculated as a function of generated resorufin. For calculation of kinetic constants, reactions were set up with varying concentration of the substrate (0-70 μM).

### Parasite culture, plasmid construct, and parasite transfection

*Plasmodium falciparum* strain 3D7 was cultured in RPMI media (Invitrogen) supplemented with 0.5% albumin and 4% haematocrit, and parasite cultures were synchronized by repeated sorbitol treatment. To generate a transfection vector construct for C-terminal DDD-HA tagging, a C-terminal fragment of *pfLPL1* gene (241-1059 bp) was amplified from *P. falciparum* 3D7 genomic DNA using primers 971A (5′-*CTCGAG*ATATATGAAGGTAGTTGGATTG-3′) and 972A (5′ *CCTAGG*TTCACATTTTTTTATCCAAG-3′). The amplified PCR product was digested with *Xho*I and *Avr*II restriction enzymes and cloned in frame to the N-terminus of GFP in the *Xho*I and *Avr*II sites of the vector pGDB [21] to yield construct pGDB-*Pf*LPL1. The plasmids were transfected into *P. falciparum* Plasmepsin-I knockout parasite line (PM1 KO), which contains the human DHFR (hDHFR) integrated into a non-essential gene [21]. Synchronized ring-stage parasites were transfected with 100 μg of purified plasmid DNA (Plasmid Midi Kit, Qiagen, Valencia, CA) by electroporation (310 V, 950 μF) [63]. Transfected parasites were selected over 2.5 μg/ml blasticidin (Calbiochem) and 5 μM trimethoprim (TMP) (Sigma) and subsequently subjected to on and off cycling of blasticidin drug to promote integration of the plasmid in the main genome. Integration was assessed by PCR-based analysis, and parasites with integration were obtained after two rounds of blasticidin cycling. The TMP was always present in the medium after its initial introduction. To generate a transfection vector construct for C-terminal HA-DD-tagging, a C-terminal fragment of *pfLPL1* gene (241-1059 bp) was cloned in XhoI and AvrII restriction sites of the vector pHADD [37, 38] inframe with HA-DD cassette to yield construct pHADD-LPL1. Transfected parasites were selected over 2.5nM WR99210 drug and 1 μM Shield1; selected parasites subsequently subjected to on and off cycling of WR99210 drug to promote integration of the plasmid in the main genome.

### Isolation of parasites and Western immunoblotting

For Western blot analyses, parasites were isolated from tightly synchronized cultures at trophozoite stage by lyses of infected erythrocyte with 0.15% saponin. Parasite pellets were washed with PBS, suspended in Laemmli buffer, boiled, and centrifuged, and the supernatant obtained was resolved on 12% SDS-PAGE. The fractionated proteins were transferred from the gel onto a PVDF membrane (Amersham) and the membrane was blocked in blocking buffer (1 × PBS, 0.1% Tween-20, 5% milk powder) for 2 h. The blot was washed and incubated for 1 h with primary antibody [mice anti-GFP (1:1000, Roche-Sigma Aldrich); rabbit anti-Bip (1:2000) [64], rabbit anti-SERA (1:2000) [65]] diluted in dilution buffer (1× PBS, 0.1% Tween-20 and 1% milk powder). Later, the blot was washed and incubated for 1 h with appropriate secondary antibody (anti-rabbit or anti-mouse, 1:2000, Sigma Aldrich) conjugated to HRP, diluted in dilution buffer. Bands were visualized by using HRP-DAB_._

### Fluorescence microscopy and indirect immunofluorescence assay

*P. falciparum* culture transfected with pGDB-*Pf*LPL1 (labelled as *Pf*LPL1-GFP-DDD) was synchronized by two sorbitol treatments 4 h apart. Parasites at different developmental stages were collected from the culture for fluorescence microscopy and stained with DAPI at a final concentration of 2 μg/ml for 30 min at 37 °C prior to imaging. Fluorescence from DAPI and GFP was observed and captured from live cells within 30 min of mounting the under a cover slip on a glass slide, using Nikon A1 confocal laser scanning microscope.

To visualize the endoplasmic reticulum, the transgenic parasites were stained with ER-Tracker Red CMXRos (Invitrogen) at a final concentration of 20 nM in 1× PBS for 15 min at 37 °C. The membrane structure in parasitized erythrocytes was labelled with BODIPY-TR-ceramide (Invitrogen) [40, 66]. Briefly, parasitized erythrocytes were resuspended in complete media (5% parasitaemia, 4% haematocrit) and incubated with 1 μM BODIPY-TR-ceramide (Invitrogen) at 37 °C for 60 min, washed in complete media three times, and examined by fluorescence microscopy. To label the neutral lipid storage structures, infected erythrocytes were labelled with Nile Red (Molecular Probes) using a modified method of Palacpac et al. [60]. Briefly, Nile Red was added to a final concentration of 1 μg/ml into the parasite culture (5–10% parasitaemia, 3% haematocrit); the cultures were incubated on ice for 30 min and subsequently washed with 1 × PBS before analysis by confocal microscopy.

Indirect immunofluorescence assays were performed on *P. falciparum* 3D7 or transgenic parasite lines as described earlier [67]. Briefly, the parasite samples were fixed with 4% paraformaldehyde, incubated with primary antibody (rabbit anti-SERA diluted 1:500 in 3% BSA, 1× PBS; rabbit anti-HA (Sigma) diuted 1:1000 in 3% BSA, 1× PBS ) and subsequently with Alexa 594 linked goat anti-rabbit antibodies (1:250, Life Technologies, USA) as secondary antibody with intermittent washing. The parasite nuclei were stained with DAPI (2 μg/ ml, Sigma Aldrich).

The GFP expressing parasites as well as the parasite stained with different labelling dyes were viewed using a Nikon A1 confocal laser scanning microscope. For live cells, observations were limited to 30 min to ensure parasite viability throughout the analyses. The 3D images were constructed by using series of Z-stack images using IMARIS 7.0 (Bitplane Scientific) software. The confocal microscope used has a DU4 detector system which is a narrow band pass filters system. Thus, avoids any cross talk between signals from Nile red (excitation/emissions maxima is 552/636 nm) and GFP (488/509 nm).

### Cryo-immunoelectron microscopy

Immunoelectron-microscopy was carried out on transgenic *P. falciparum* parasites, *Pf*LPL1-GFP-DDD, expressing *Pf*LPL1 fused with GFP tag. The trophozoite stage parasites were fixed in 4% paraformaldehyde and 0.04% glutaraldehyde in 1× PBS at 4 °C for 1 h and subsequently embedded in gelatin and infiltrated with a cryo-preservative and plasticizer (2.3 M sucrose/20% polyvinyl pyrrolidone). After freezing in liquid nitrogen, samples are sectioned with a Leica Ultracut UCT cryo-ultramicrotome (Leica Microsystems, Bannockburn, IL) at − 260 °C. Ultra-thin sections were blocked with 5% foetal bovine serum and 5% normal goat serum in 1× PBS for 30 min and subsequently stained with rabbit anti-GFP antibody (Abcam, 1:500 dilution in blocking buffer), washed thoroughly, and incubated with 18 nm colloidal gold-conjugated anti-rabbit IgG for 1 h. Sections were stained with 0.3% uranyl acetate/1.7% methyl cellulose and visualized under a JEOL 1200EX transmission electron microscope (JEOL USA, Peabody, MA). All labelling experiments were conducted in parallel with controls omitting the primary antibody or using pre-immune sera as primary antibodies.

### In vitro parasite growth assays

For growth assays, tightly synchronized parasites at ring stage were cultured in a 6-well plate. Each well contained 4% haematocrit and 1 ml of complete media supplemented with 5 μM TMP, and the parasitaemia was adjusted to 0.5% by diluting the parasite cultures with appropriate amount of uninfected erythrocytes (4% haematocrit). A parallel set of parasite culture was grown in media without TMP. For microscopic analysis, smears were made from each well at different time points (0, 24, 48, 72, 96, and 120 h) stained with Giemsa, and the numbers of ring/trophozoite stage parasites per 5000 RBCs were determined and percentage parasitaemia [(number of infected erythrocytes/total number of erythrocytes) × 100] was calculated to assess the parasite inhibition. Each of the assay was performed three times separately on different days.

For fatty acid/lipid complementation assay, tightly synchronized ring stage parasite cultures (6 ± 2 hpi) were supplemented with fatty acids (30 μM palmitic acid [C16:0; Sigma] and 45 μM oleic acid [C18:1; Sigma] or 20 μM LPC 16:0 (Sigma) and 20 μM LPC18:1 (Sigma). Parasites were allowed to develop until the trophozoite stage (30 ± 2 hpi) or ring stage (6 ± 2 hpi) for hemozoin assay and growth assay respectively. Parasites were harvested at indicated time points. All assays were performed in triplicates and on different days.

### Lipid isolation and mass spectrometry-based lipidomic analyses

Infected red blood cells (5 × 10^7^ cell equivalents) were harvested from control *Pf*LPL1-iKO cultures and parasites released from the erythrocyte hosts by saponin treatment as previously described [6]. The parasite pellets were washed in PBS and stored at − 80 °C or directly used for further analysis. Their total lipid spiked with 20 nmol C21:0 phosphatidylcholine was extracted by chloroform to methanol, 1:2 (v/v), and chloroform to methanol, 2:1 (v/v). The pooled organic phase was subjected to biphasic separation by adding 0.1% KCl and was then dried under N_2_ gas flux prior to being dissolved in 1-butanol. For the total fatty acid analysis, an aliquot of the lipid extract was derivatized on-line using MethPrep II (Alltech), and the resulting FA methyl esters were analysed by GC-MS as previously described [8]. For the quantification of each lipid, total lipid was separated by 2D HPTLC using chloroform/methanol/28% NH_4_OH, 60:35:8 (v/v/v) as the 1st dimension solvent system and chloroform/acetone/methanol/acetic acid/water, 50:20:10:13:5 (v/v/v/v/v), as the 2nd dimension solvent system. Each lipid spot was extracted for quantification of fatty acids by gas chromatography-mass spectrometry (Agilent 5977A-7890B) after methanol lysis. Fatty acid methyl esters were identified by their mass spectrum and retention time and quantified by Mass Hunter Quantification Software (Agilent), and the calibration curve generated with fatty acid methyl esters standards mix (Sigma CRM47885). Then, each lipid content was normalized according to the parasite cell number and a C21:0 internal standard (Avanti Polar lipids). All analyses were performed in triplicate or more (*n* = 3). * *P* values of ≤ 0.05 from statistical analyses (Student’s *t* test) obtained from GraphPad software analysis were considered statistically significant.

### Hemozoin isolation and estimation

Total hemozoin was purified following Coban et al. [68]*.* Briefly, equal number of parasitized erythrocytes with trophozoite stage parasites were collected from synchronized *P. falciparum* cultures. Parasites were harvested by saponin lysis, washed five times with phosphate-buffered saline (PBS), and sonicated in 2% SDS. The pellet was washed seven to eight times in 2% SDS, resuspended in 10 mM Tris-HCl (pH 8.0), 0.5% SDS, and 1 mM CaCl_2_ containing proteinase-K (2 mg/ml), and incubated at 37 °C overnight. The pellet was again washed three times in 2% SDS and incubated in 6 M urea for 3 h at room temperature with shaking. The pellet was again washed three times in 2% SDS and then in distilled water. The final hemozoin pellet was resuspended in distilled water. Total heme content was determined following Tripathi et al. [69]. The hemozoin was dissolved by incubation in 1 ml 20 mM NaOH/2% SDS at room temp for 2 h. The absorbance of solution was taken at 405 nm and heme concentration was calculated from standard curve. Heme stock (10 mM) was prepared by dissolving 3.3 mg of hemin (Sigma) in 500 μl of 1 M NaOH, which was used to make dilutions ranging from 50 μM- 600 μM to plot the standard curve.

### Flow cytometry

The change in levels of GFP tagged protein and neutral lipids stored in transgenic parasites were assessed by flow cytometry. Tightly synchronized ring stages transgenic parasite cultures were grown in media containing 5 μM TMP or solvent alone; aliquot of the parasite samples were collected after 24 h. GFP fluorescence and Nile Red staining was quantified using the BD FACS Calibur system (Beckton Dickinson). Arbitrary fluorescence units for 100,000 cells were acquired on channels FL1 (GFP) and FL2 (Nile red) using Cell Quest Pro (Beckton Dickinson), and data were analysed using Graph Pad Prism v 5.0. Uninfected RBCs were used as background control.

### Statistical analysis

The data sets were analysed and graphical presentations were made using GraphPad Prism ver 5.0, and the data were compared using unpaired Student’s *t* test.

## Supplementary Information

**Additional file 1: Table S1.** List of putative phospholipases in *P. falciparum*, their expression pattern in different parasite stages and domain architecture. **Figure S1.** Clustal W alignment of amino acid sequences of lysophospholipase (LPL1) homologues from different species of Plasmodium. **Figure S2.** Clustal W alignment of amino acid sequences showing the conserved GXSXG motif in homologues of LPL1. **Figure S3.** Biochemical characterization of recombinant PfLPL1. **Figure S4.** Fluorescent and time-lapse microscopy to show localization as well as trafficking of P*f*LPL1-RFA fusion protein in transgenic P. falciparum parasites. **Figure S5.** Structured illumination microscopy (SIM) images of live transgenic parasites showing labelling of membranes in infected RBCs and localization of P*f*LPL1. **Figure S6.** Generation of transgenic parasites expressing P*f*LPL1 with ddFKBP degradation domain tag, PiLPLl-DD parasite line. **Figure S7.** Expression and localization of the P*f*LPLl-DD fusion protein in transgenic parasites. **Figure S8.** Inducible knock-down of P*f*LPL1 protein in the P*f*LPL1-DD transgenic parasites and its effect on growth and development of the parasites. **Figure S9.** Replicative data sets for Fig. 5C. **Figure S10.** Replicative data sets for Fig. 6A and 6B. **Figure S11.** Host-derived LPC is not able to complement P*f*LPL1 iKO.

## Data Availability

All data generated or analysed during this study are included in this published article and its supplementary information file. Source data file can be provided upon specific request to the corresponding author.
