# Supplementary material for: An essential vesicular-trafficking phospholipase mediates neutral lipid synthesis and contributes to hemozoin formation in Plasmodium falciparum
Source: BMC Biol. 2021 Aug 11;19:159. doi: 10.1186/s12915-021-01042-z (PMC8359613; doi:10.1186/s12915-021-01042-z)
Supplement: Supplementary file 1 — Additional file 1: Table S1. List of putative phospholipases in P. falciparum, their expression pattern in different parasite stages and domain architecture. Figure S1. Clustal W alignment of amino acid sequences of lysophospholipase (LPL1) homologues from different species of Plasmodium. Figure S2. Clustal W alignment of amino acid sequences showing the conserved GXSXG motif in homologues of LPL1. Figure S3. Biochemical characterization of recombinant PfLPL1. Figure S4. Fluorescent and time-lapse microscopy to show localization as well as trafficking of PfLPL1-RFA fusion protein in transgenic P. falciparum parasites. Figure S5. Structured illumination microscopy (SIM) images of live transgenic parasites showing labelling of membranes in infected RBCs and localization of PfLPL1. Figure S6. Generation of transgenic parasites expressing PfLPL1 with ddFKBP degradation domain tag, PiLPLl-DD parasite line. Figure S7. Expression and localization of the PfLPLl-DD fusion protein in transgenic parasites. Figure S8. Inducible knock-down of PfLPL1 protein in the PfLPL1-DD transgenic parasites and its effect on growth and development of the parasites. Figure S9. Replicative data sets for Fig. 5C. Figure S10. Replicative data sets for Fig. 6A and 6B. Figure S11. Host-derived LPC is not able to complement PfLPL1 iKO. [file 12915_2021_1042_MOESM1_ESM.pdf]

Table S1: List of putative phospholipases in *P. falciparum*, their expression pattern in different parasite stages and domain architecture

PlasmoDB Gene ID	Expression in parasite stages*	Functional Domains by PFAM/SSDB Motif Search (Functional annotation)	Domain length (aa)	Pfam E-value	Protein name (Reference or this study)
PF3D7_1476700	R, Sch, G	α/β hydrolase, (putative lysophospholiapase)<br>Prolyl oligopeptidase	86-337<br>259-352	2.8e-28<br>6e-06	PfLPL1
PF3D7_1038900	G-V, Oo	α/β hydrolase, putative esterase	85-340	8.1e-29	PfLPL2
PF3D7_1476800	R to Sch, G	α/β hydrolase, putative lysophospholiapase<br>Prolyl oligopeptidase	86-337<br>256-351	2.4e-29<br>7.1e-05	PfLPL3
PF3D7_0731800	R, G, Oo	α/β hydrolase	290-503	2.6e-22	PfLPL4
PF3D7_0936700	R , Sch	α/β hydrolase, putative lysophospholipase	90-141, 226-405	6.4e-09<br>3.5e-14	PfLPL10
PF3D7_0702200	R &Sch, G-V	α/β hydrolase, putative lysophospholipase	86-234, 206-385	1.3e-08<br>2e-17	PfLPL20
PF3D7_1252600	R to Sch	α/β hydrolase, putative lysophospholipase	90-140, 171-347	2.4e-07<br>6.2e-16	PfLPL30
PF3D7_1401500	R, T, G	α/β hydrolase, putative lysophospholipase	90-140, 153-345	2.5e-08<br>3.4e-13	PfLPL40
PF3D7_0102400	G-V	α/β hydrolase, putative lysophospholipase, pseudogene	43-350	6.38e-30	PfLPL50
PF3D7_0709700	G-V	α/β hydrolase, putative lysophospholipase	95-344	7.1e-28	PfLPL60
PF3D7_0937200	R, G	α/β hydrolase, putative lysophospholipase	85-338	4.6e-28	PfLPL70
PF3D7_1001400	R	α/β hydrolase, exported lipase 1, lysophospholipase/serine aminopeptidase	550-767	5.7e-18	PfXL1#
PF3D7_1001600	T-Sch	α/β hydrolase, (exported lipase 2, lysophospholipase/serine aminopeptidase)	390-527	4.56e-17	PfXL2#
*R-ring, T-trophozoite, Sch-schizont, G-gametocyte, Oo-ookinete stages			#Spillman *et al.*, [19]		

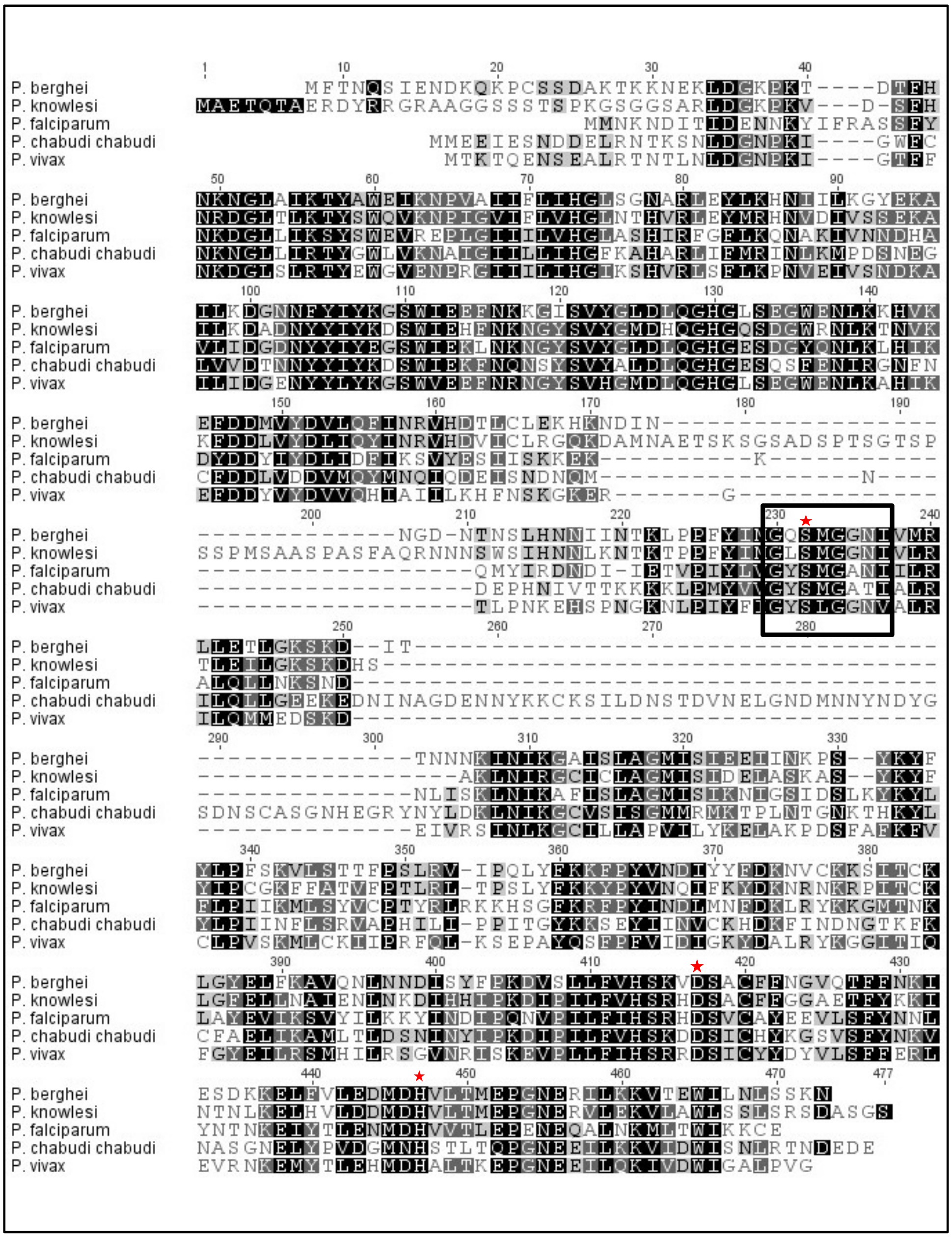

Figure S1

Figure S1: Clustal W alignment of amino acid sequences of lysophospholipase (LPL1) homologues from different species of *Plasmodium*. Amino acids with 100% identity are shown in black, ≥80% in dark grey and ≥60% in light grey. The conserved GXSXG motif is boxed and the conserved catalytic residues (S, D and H) are marked with asterisks.

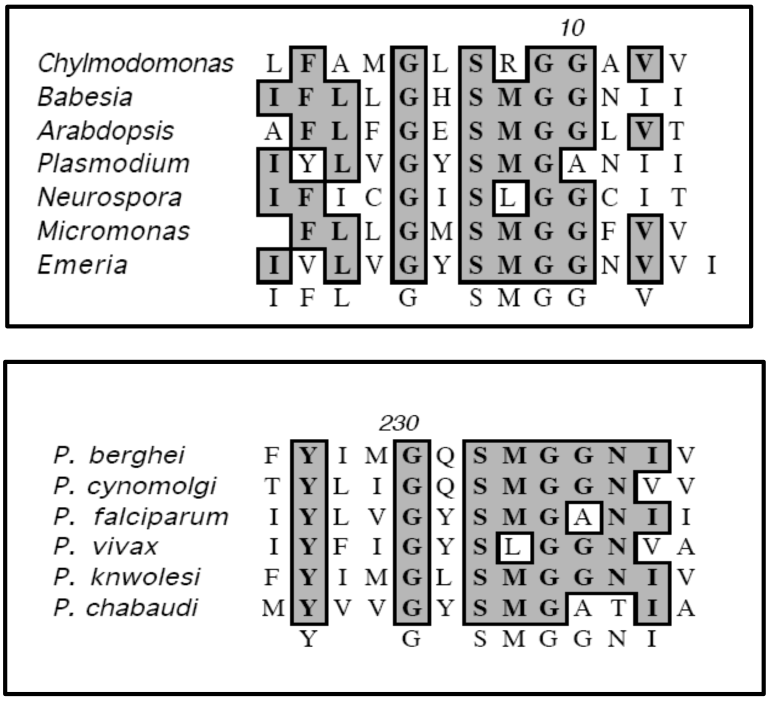

Figure S2

Figure S2: Clustal W alignment of showing the conserved GXSXG motif in homologues of LPL1 of *Emeria tenella* (AET50683.1)*, Micromonas sp. RCC299* (XP_002500615.1)*, Neurospora caninum liverpool (XP_003881427.1) , Arabidopsis thaliana (NP_175685.1), Babesia equi* (XP_004833376.1)*, Chylmodomonas reinhardtii* (XP_001690806.1) *and Plasmodium species*. Amino acids with ≥60% identity are shown in grey.

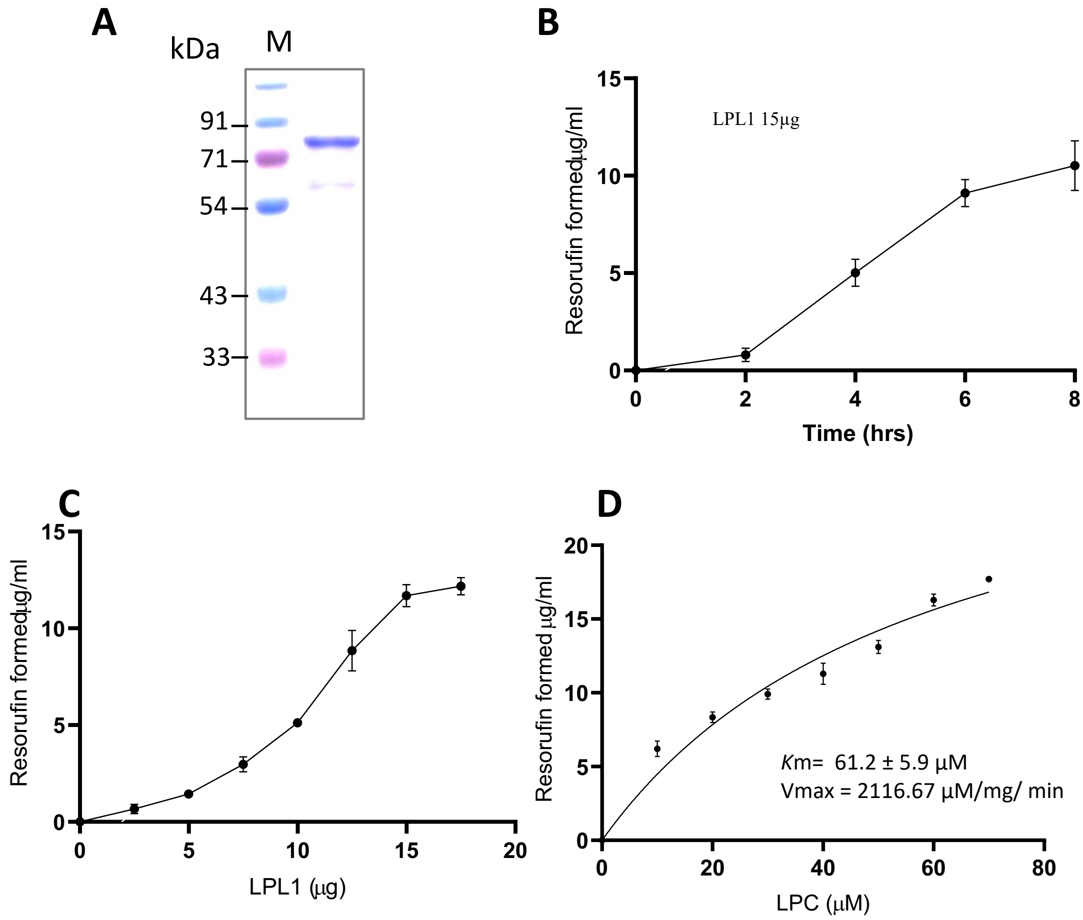

Figure S3: Biochemical characterization of recombinant *Pf*LPL1: (A) Expression and purification of recombinant PfLPL1 as MBP-3xHis fusion protein. The full gene *pflpl1* was cloned into pETM-41 vector and recombinant protein was expressed in BL21 (DE3) *E. coli* cells. It was purified by two- step affinity chromatography using Ni^{2+}-NTA and amylose resins. SDS-PAGE showing purified recombinant protein (~72kDa). (B-D) *In vitro* activity of recombinant *Pf*LPL1 was assessed by choline release assays using LPC as substrate and Amplex-Red detection kit. (B) Time dependent *Pf*LPL1 activity using 15µg of recombinant protein in the assay reaction. (C) *Pf*LPL1 activity at different protein concentrations (2.5 - 17.5µg) at 6h time point. (D) Line graph showing Michaelis-Menten fit curve developed for *Pf*LPL1 activity using different concentrations of LPC substrate.

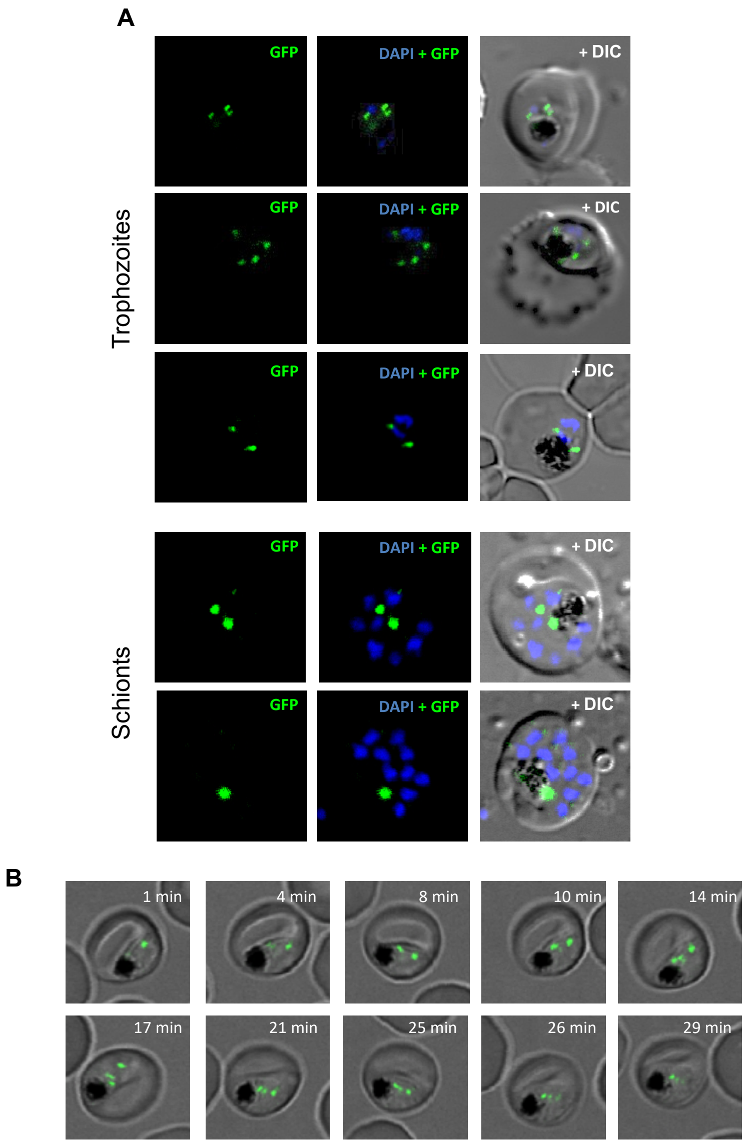

Figure S4: (A) Localization of *Pf*LPL1-RFA fusion protein in transgenic *P. falciparum* parasites. Fluorescent microscopic images of live transgenic parasites trophozoite, and stages. The parasite nuclei were stained with DAPI and slides were visualized by confocal laser scanning microscope. The GFP-fluorescence was observed in small vesicles present near the parasitophorous vacuole and in the parasite cytosol. In late trophozoite stage GFP-fluorescence was observed in large vesicular structure in close association with the food-vacuole. **(B)** Consecutive images from time-lapse microscopy of *Pf*LPL1-RFA expressing transgenic parasites showing localization and migration GFP labeled vesicles. Sequential images of a parasite over a time interval of 29 min showing development of a GFP labeled vesicles at the parasite plasma membrane, and series of vesicles traversing in parasite cytosol towards food-vacuole (labelled by presence of hemozoin crystals).

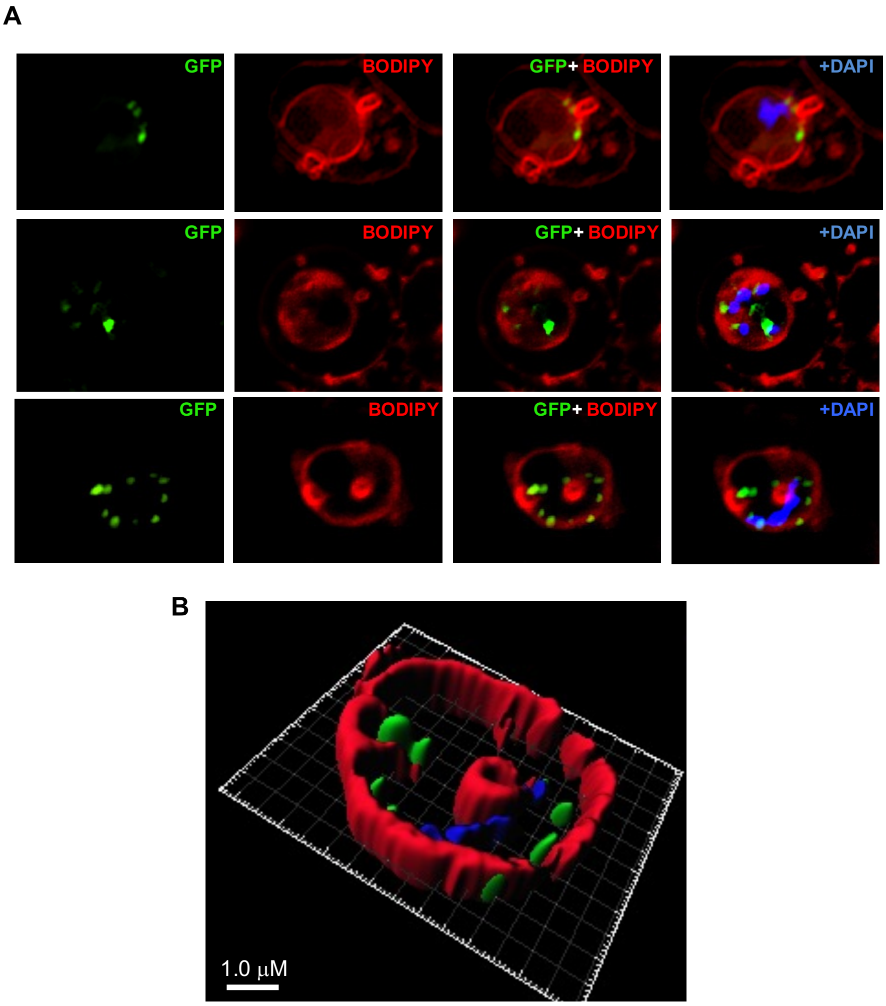

Figure S5: Structured illumination microscopy (SIM) images of live transgenic parasites showing labelling of membranes in infected RBCs and localization of *Pf*LPL1. (A) Trophozoite stage transgenic parasites expressing *Pf*LPL1-RFA were stained with BODIPY-TR ceramide (red) and parasite nuclei were stained with DAPI (blue). Small GFP foci of the *Pf*LPL1-RFA fusion protein were observed near parasite boundary (panel 1, marked with arrowhead), these foci showed closed association with fluorescence by BODIPY-TR labelled parasite membrane. In some parasites, the GFP vesicles are seen in parasite cytosol (panel 2 and 3). **(B)** A three-dimensional reconstruction of series of Z-stack images (corresponding to panel 3 in A) using IMARIS software. Small GFP vesicles are present juxtaposed to the parasite membrane and in parasite cytosol.

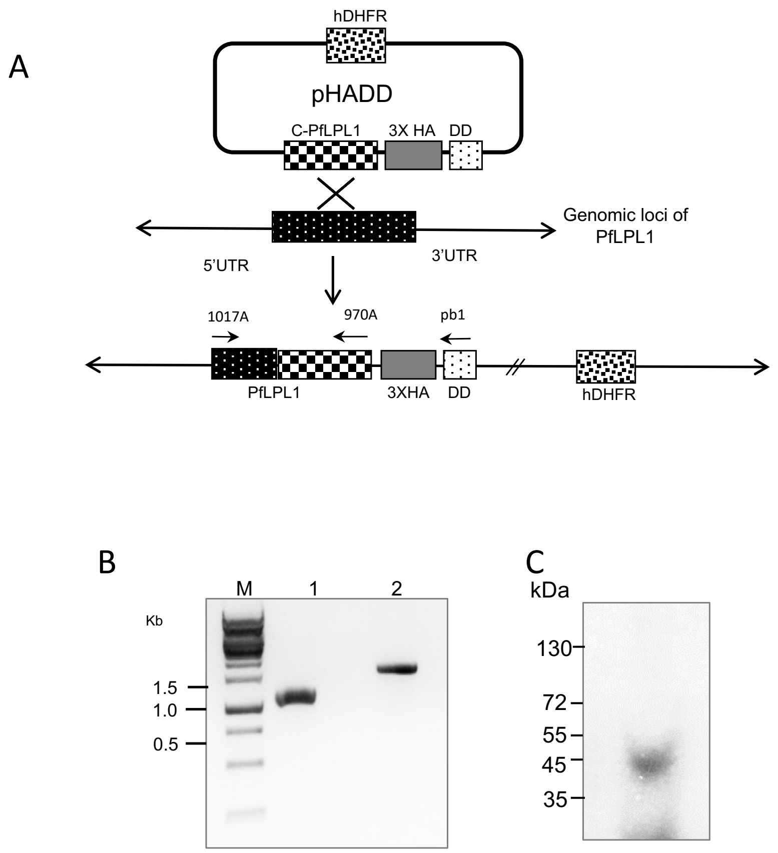

Figure S6: Generation and analysis of *Pf*LPLl-DD parasite line. (A) Schematic of pHADD vector illustrating the targeting plasmid for integration in the *Pf*LPLl locus. A fragment corresponding to the 3' end of the *pfLPL1* gene was cloned upstream of 3XHA tag and ddFKBP gene in the pHADD vector. The primer combinations used PCR analysis to confirm the integration are indicated. **(B)** PCR amplification using genomic DNA from the *Pf*LPLl-DD parasite lines to confirm the integration of the plasmid in the main genome locus. using the primer combination 1017A/970A (lane 1) and 1017A/Pb1 (lane 2). **(C)** Immunoblot analysis of trophozoite stage *Pf*LPLl-DD transgenic parasites grown in presence of Shld1 drug, using anti-HA antibody. A band of ~ 53 kDa, representing the fusion protein, is detected in transgenic parasites.

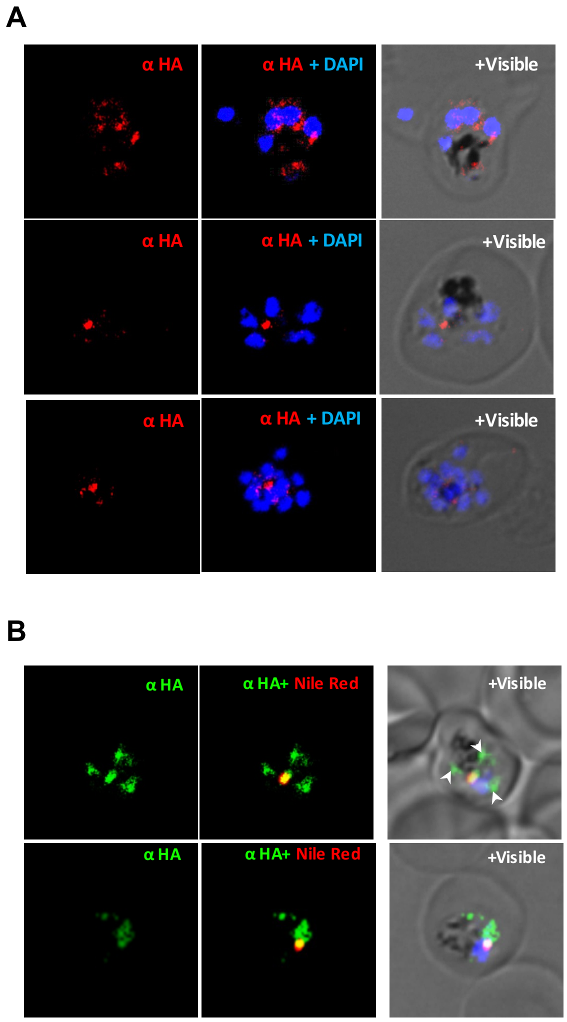

Figure S7: Expression and localization of the *Pf*LPLl fusion protein with ddFKBP degradation domain (*Pf*LPLl-DD) in transgenic parasites. (A) Fluorescence microscopy images of *Pf*LPLl-DD transgenic parasite immuno-stained with anti-HA antibody. **(B)** Fluorescence microscopy images of *Pf*LPLl-DD transgenic parasite immuno-stained with anti-HA antibody and co-stained with Nile red . The parasite nuclei were stained with DAPI and slides were visualized by confocal microscope. The *Pf*LPL1 was localized in vesicular structures which overlapped in neutral lipid body in late trophozoite stages

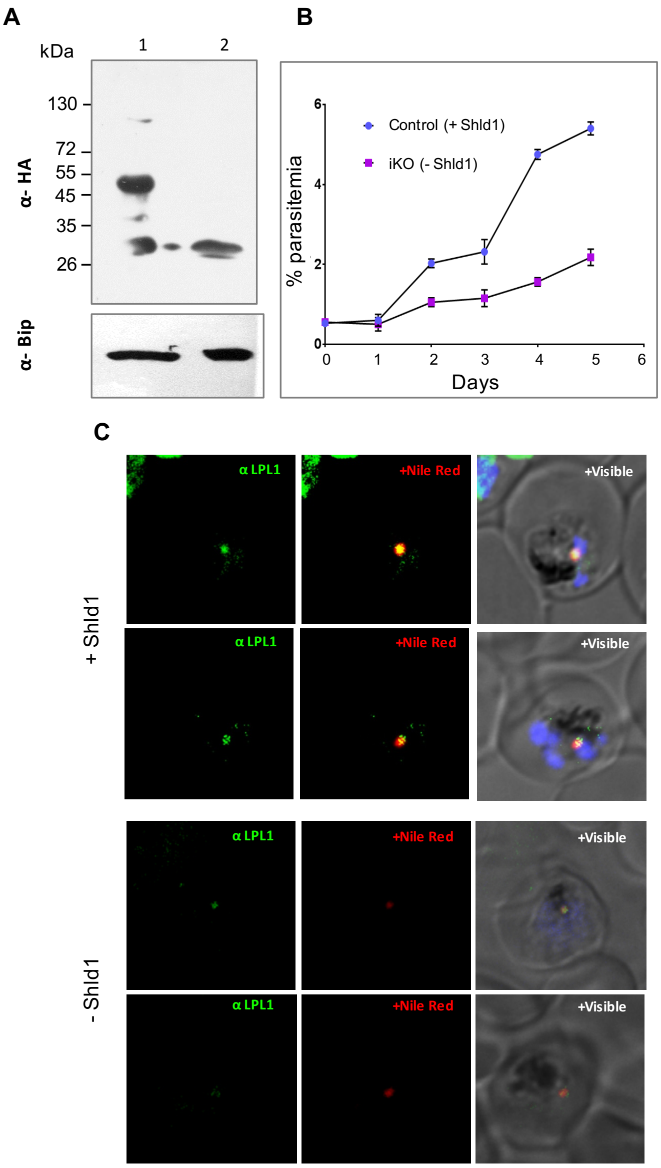

Figure S8: Inducible knock-down of *Pf*LPL1 protein in the *Pf*LPL1-DD transgenic parasites and its effect on growth and development of the parasites. (A) Immunoblot analysis using anti-HA antibodies and trophozoite stage transgenic parasites expressing *Pf*LPL1-DD grown in presence of Shld1 drug or solvent alone (control or iKO respectively). A band of ~ 53 kDa, representing the fusion protein, is detected in the control parasites (lane 1), but not in the *Pf*LPL1-iKO set (lane 2). Parallel blot was probed with anti-Bip antibodies to show equal loading. **(B)** Tightly synchronized ring stage parasite culture (0.2% parasitemia) of transgenic parasites were grown with or without Shld1 (control and *Pf*LPL1-iKO, respectively), and their growth was

monitored for three cycles by estimating total parasitemia at 48, 96 and 144h. **(C)** Effect of inducible knock-down of *Pf*LPL1 protein in the *Pf*LPL1-DD transgenic parasites on the development of neutral lipid body. Synchronous transgenic parasites at ring stages were grown till late trophozoite stages in presence or absence of Shld1 (control and iKO respectively) immuno-stained with anti-*Pf*LPLl antibody and stained with Nile red. Fluorescence images of trophozoites stage transgenic parasites in *Pf*LPL1-iKO set, showing reduction in Nile red fluorescence intensity and loss of *Pf*LPLl as compared to control parasite. The parasite nuclei were stained with DAPI (blue) and parasites were visualized by confocal laser scanning microscope.

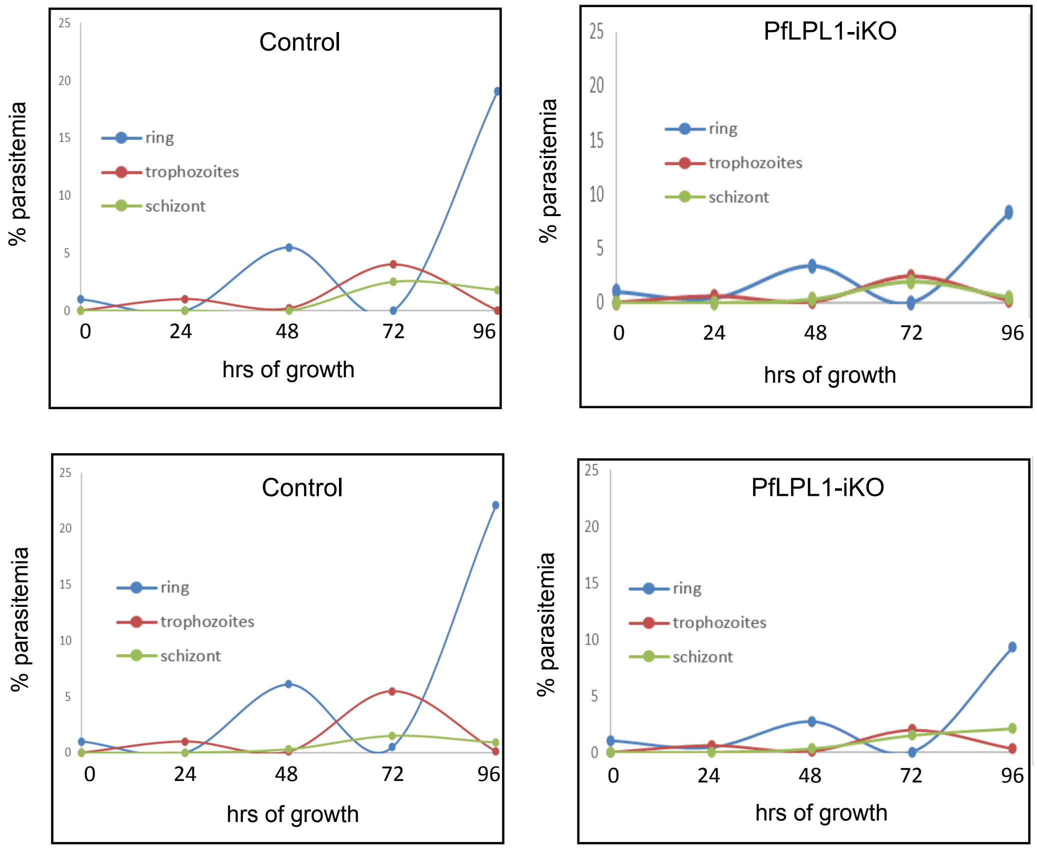

Figure S9: Replicative data sets for Figure 5C; Graphs showing parasite stage composition at different time points (0-120h) in parasite culture from control and *Pf*LPL1-iKO sets.

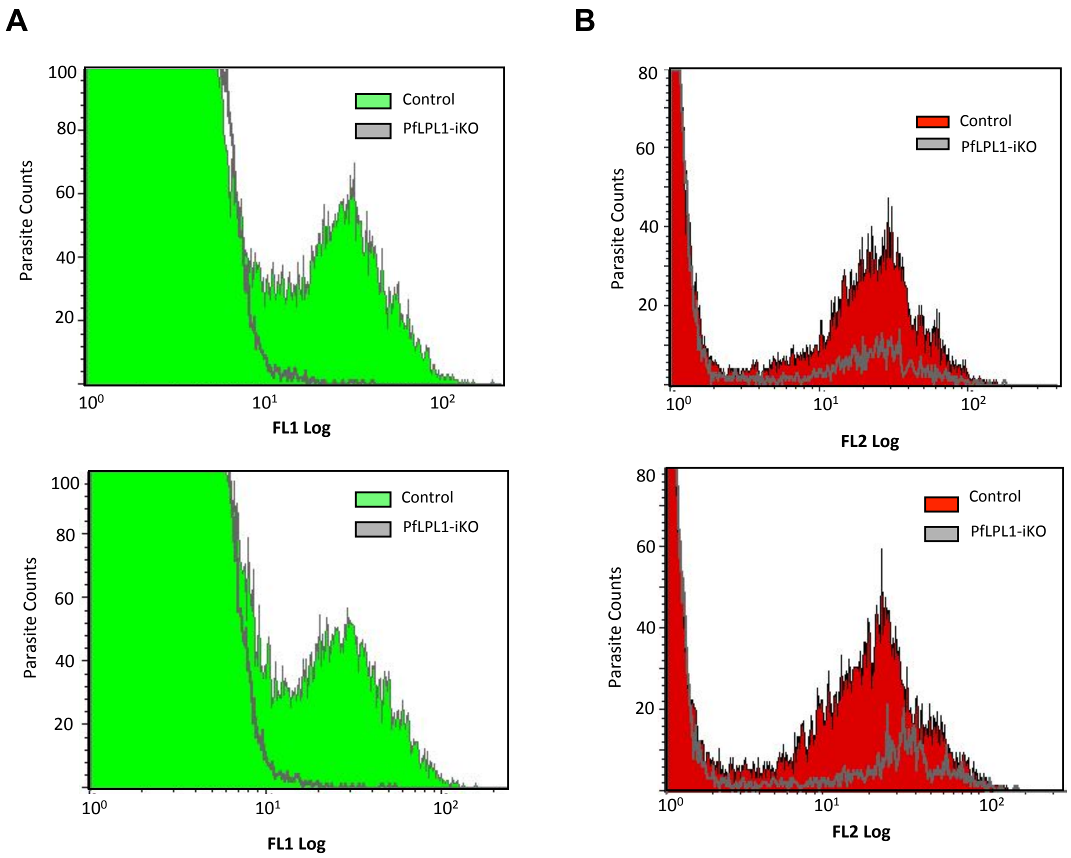

Figure S10: Replicative data sets for Figure 6A and 6B; Synchronous transgenic parasites at ring stages were grown till late trophozoite stages in control and *Pf*LPL1-iKO sets, stained with Nile red and analysed by flow cytometry. Flow cytometry histogram showing concomitant reduction in GFP fluorescence (FL-1) (A) and Nile red labelling (FL-2) (B) in parasites after *Pf*LPL1-iKO as compared to control parasites.

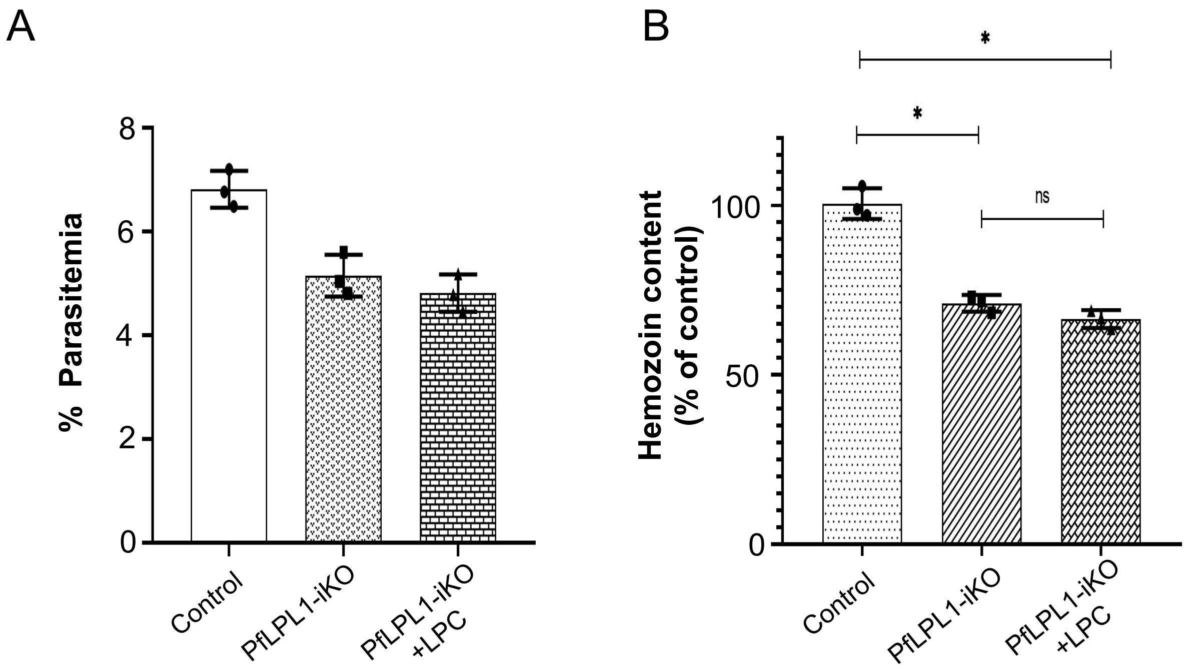

Figure S11: Host-derived LPC is not able to complement *Pf*LPL1 iKO. Synchronous transgenic parasites in the *Pf*LPL1-iKO set grown in presence or absence of LPC (18:1), the parasite growth as well as total hemozoin content was assessed as compared to control. (A) Graph showing % parasitemia in *Pf*LPL1-iKO+LPC set as compared to *Pf*LPL1-iKO. (B) Graph showing hemozoin content in *Pf*LPL1-iKO+LPC set as compared to *Pf*LPL1-iKO. The *p* values were calculated by Student's t-test: * p <0.01
